# The Molecular Mechanism of Nitrate Chemotaxis via Direct Ligand Binding to the PilJ Domain of McpN

**DOI:** 10.1128/mBio.02334-18

**Published:** 2019-02-19

**Authors:** David Martín-Mora, Álvaro Ortega, Miguel A. Matilla, Sergio Martínez-Rodríguez, José A. Gavira, Tino Krell

**Affiliations:** a Estación Experimental del Zaidín, Department of Environmental Protection, Consejo Superior de Investigaciones Científicas, Granada, Spain; b Departamento de Bioquímica y Biología Molecular III e Inmunología, Universidad de Granada, Melilla, Spain; c Laboratorio de Estudios Cristalográficos, IACT, Superior de Investigaciones Científicas (CSIC) y la Universidad de Granada (UGR), Armilla, Spain; Massachusetts Institute of Technology

**Keywords:** *Pseudomonas aeruginosa*, chemoreceptor, chemotaxis, nitrate

## Abstract

Chemotaxis and energy taxis permit directed bacterial movements in gradients of environmental cues. Nitrate is a final electron acceptor for anaerobic respiration and can also serve as a nitrogen source for aerobic growth. Previous studies indicated that bacterial nitrate taxis is mediated by energy taxis mechanisms, which are based on the cytosolic detection of consequences of nitrate metabolism. Here we show that Pseudomonas aeruginosa PAO1 mediates nitrate chemotaxis on the basis of specific nitrate sensing by the periplasmic PilJ domain of the PA2788/McpN chemoreceptor. The presence of nitrate reduced *mcpN* transcript levels, and McpN-mediated taxis occurred only under nitrate starvation conditions. In contrast to the NarX and NarQ sensor kinases, McpN bound nitrate specifically and showed no affinity for other ligands such as nitrite. We report the three-dimensional structure of the McpN ligand binding domain (LBD) at 1.3-Å resolution in complex with nitrate. Although structurally similar to 4-helix bundle domains, the ligand binding mode differs since a single nitrate molecule is bound to a site on the dimer symmetry axis. As for 4-helix bundle domains, ligand binding stabilized the McpN-LBD dimer. McpN homologues showed a wide phylogenetic distribution, indicating that nitrate chemotaxis is a widespread phenotype. These homologues were particularly abundant in bacteria that couple sulfide/sulfur oxidation with nitrate reduction. This work expands the range of known chemotaxis effectors and forms the basis for the exploration of nitrate chemotaxis in other bacteria and for the study of its physiological role.

## Abstract (precis)

Nitrate is of central importance in bacterial physiology. Previous studies indicated that movements toward nitrate are due to energy taxis, which is based on the cytosolic sensing of consequences of nitrate metabolism. Here we present the first report on nitrate chemotaxis. This process is initiated by specific nitrate binding to the periplasmic ligand binding domain (LBD) of McpN. Nitrate chemotaxis is highly regulated and occurred only under nitrate starvation conditions, which is helpful information to explore nitrate chemotaxis in other bacteria. We present the three-dimensional structure of the McpN-LBD in complex with nitrate, which is the first structure of a chemoreceptor PilJ-type domain. This structure reveals striking similarities to that of the abundant 4-helix bundle domain but employs a different sensing mechanism. Since McpN homologues show a wide phylogenetic distribution, nitrate chemotaxis is likely a widespread phenomenon with importance for the life cycle of ecologically diverse bacteria.

## INTRODUCTION

Many bacteria are capable of flagellum-driven tactic movements in stimulus gradients. Genome analyses have revealed that more than half of the bacterial genomes contain genes necessary for taxis (1). The canonical form of taxis is based on stimulus reception by the chemoreceptor that leads to changes in the activity of the CheA autokinase, which subsequently modulates transphosphorylation to the CheY response regulator. The phosphorylated form of CheY binds to the flagellar motor, ultimately causing taxis toward or away from the stimulus (2).

Many bacteria possess the capacity to perform chemotaxis and energy taxis. The typical chemotaxis mechanism is initiated by the recognition of specific chemoeffectors at periplasmic ligand binding domains (LBDs), leading to receptor activation. Chemoreceptor function is largely determined by the nature of the chemoeffectors recognized, and the chemoreceptors specific for different compound classes such as amino acids, tricarboxylic acid (TCA) cycle intermediates, polyamines, purines, or inorganic phosphate have been identified (3). Alternatively, energy taxis is a metabolism-dependent form of taxis and represent directed movement in gradients of physicochemical parameters that affect metabolism (4). In contrast to chemotaxis, it is not the chemoeffector that is sensed *per se* but the consequences of its metabolism. Energy taxis occurs in response to a very wide range of stimuli, including metabolizable substrates such as sugars, organic acids, and amino acids; electron acceptors such as oxygen, nitrate, fumarate, and dimethyl sulfoxide; and compounds that affect metabolism otherwise, such as light or metabolic inhibitors (5–7).

Escherichia coli, the model organism traditionally used to study chemotaxis, has 4 chemoreceptors with a periplasmic LBD that mediate chemotaxis primarily with respect to amino acids, sugars, or dipeptides (8). In addition, it has an Aer chemoreceptor that mediates energy taxis by sensing redox changes via a flavin adenine dinucleotide (FAD)-containing cytosolic PAS domain (8). However, genome analyses have indicated that many other bacteria have significantly more chemoreceptors (up to 80) than E. coli (5). In addition, these chemoreceptors are characterized by diversity in the LBD type since more than 80 different LBD types were found to form part of chemoreceptors (9).

The elevated number of chemoreceptors and their diversity in the LBDs suggest that the chemosensory capacity of many bacteria is very extensive but remains in general largely unexplored. The scientific community is now only at the beginning of the process of identifying the chemotactic spectra of many bacteria, establishing links between chemoeffectors and LBD types, and identifying the physiological relevance of chemotaxis to newly identified chemoeffectors (9).

Here, we have addressed this issue using the opportunistic human pathogen Pseudomonas aeruginosa PAO1 as a model (9, 10). This bacterium has 26 chemoreceptors that feed into four chemosensory pathways (11). Two pathways, corresponding to Che and Che2, were shown to play a role in chemotaxis. Whereas the Che pathway appears to be essential for chemotaxis (12, 13), the Che2 pathway was found to be required for optimal chemotactic responses (14). Alternatively, the Chp pathway was associated with twitching motility (15–18), whereas the Wsp pathway modulates cyclic diguanylate monophosphate (c-di-GMP) levels (19). P. aeruginosa chemoreceptors employ together 11 different LBD types for signal sensing (9), with the 4-helix bundle (4HB) (20), CACHE (21), and helical bimodular (HBM) (22) domains being most abundant (9). Several of these receptors have been functionally annotated, and all were found to mediate chemoattraction. The 4HB domain containing receptor CtpH responded specifically to inorganic phosphate (P_i_) (23, 24), the HBM domains containing McpK and CtpL were identified as α-ketoglutarate (25)- and P_i_ (23, 24)-specific receptors, and the paralogous dCACHE domains containing receptors PctA, PctB, and PctC were shown to mediate chemotaxis to amino acids (26–29), whereas the sCACHE domain containing receptor PA2652 was found to mediate taxis to different C4-carboxylic acids (30, 31).

P. aeruginosa has two chemoreceptors, PA2788 and PA0411/PilJ, that possess a PilJ-type LBD (Pfam signature PF13675) (9). This domain is annotated in the Pfam database (32) as “Type IV pili methyl-accepting chemotaxis transducer N-ter.” The PilJ receptor feeds into the Chp pathway and is not related to chemotaxis (18), whereas PA2788 was predicted to feed into the Che pathway (11). However, the ligands recognized by the PilJ and PA2788 are unknown. Bioinformatic analyses have shown that PilJ domains represent 2% of all chemoreceptor LBDs (9). In addition, a search in the Pfam database revealed that PilJ domains are also employed by other bacterial sensor proteins such as sensor kinases, diguanylate cyclases, and transcriptional regulators. In this study, we aimed at identifying the function of the PilJ domain containing chemoreceptors in P. aeruginosa.

## RESULTS

### Nitrate is a specific ligand for PA2788-LBD.

To identify ligands that bind to PA2788, we cloned the DNA sequence encoding the LBD of PA2788 into an expression vector. Protein was expressed in E. coli and purified by affinity chromatography. High-throughput ligand screening assays were then conducted using the thermal shift method (33). In this method, changes in the melting temperature (*T_m_*) of a protein, representing the midpoint of thermal protein unfolding, are recorded. The binding of ligands typically increases the *T_m_*, and shifts of greater than 2°C are considered significant. We screened 480 ligands from Biolog compound arrays PM1, PM2A, PM3B, PM4A, and PM5 that served as bacterial carbon, nitrogen, phosphorus, and sulfur sources.

**FIG 1:**
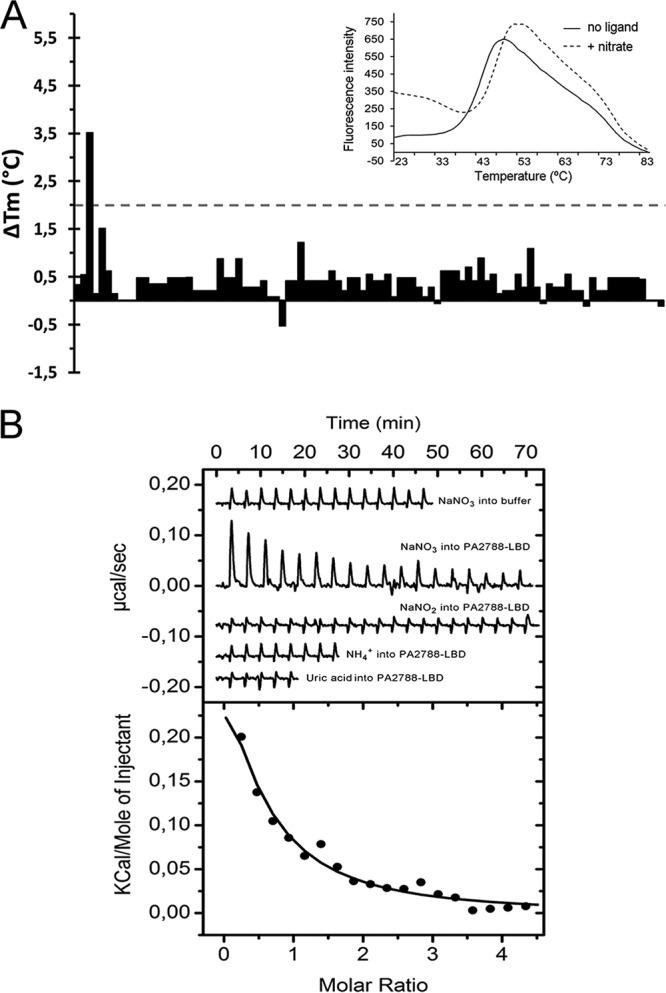
Identification of nitrate as a PA2788-LBD ligand. (A) Thermal shift assays using compounds of Biolog compound array PM3B. Shown are the *T_m_* changes with respect to the ligand-free protein. The insert shows the thermal unfolding curves of ligand-free PA2788-LBD and in the presence of nitrate. (B) Microcalorimetric binding studies of PA2788-LBD. The upper panel shows the heat changes caused by the injection of 2 mM (12.8-µl aliquots) NaNO_3_ into buffer and 36 µM PA2788-LBD as well as the titration of PA2788-LBD with 2 mM NaNO_2_, 2 mM ammonia, and 1 mM uric acid. The lower panel depicts the concentration-normalized and dilution heat-corrected integrated peak areas of the PA2788-LBD titration data with NaNO_3_. The line corresponds to the best fit using the “One binding site model” of the MicroCal version of ORIGIN.

In the absence of ligand, the *T_m_* of PA2788-LBD was 43.5°C. Fig. 1A shows the changes in *T_m_* caused by each of the 95 compounds of array PM3B comprising nitrogen sources. In the presence of NaNO_3_, the *T_m_* was increased by 3.5°C, whereas NaNO_2_ caused only a minor increase of 0.5°C. No significant *T_m_* shifts were obtained for the compounds in arrays PM1, PM2A, PM4A, and PM5.

To verify ligand binding, isothermal titration calorimetry (ITC) experiments were conducted. In an initial control experiment, the heat changes derived from the injection of 2 mM NaNO_3_ into buffer were recorded (Fig. 1B), with results showing that the levels of dilution heat were low and uniform. Titration of PA2788-LBD with the same ligand caused endothermic heat changes (Δ*H *=* *0.38 ± 0.1 kcal/mol) indicative of an entropy-driven binding process (*T*Δ*S *=* *6.4 kcal/mol) characterized by a dissociation constant of 47 ± 8 µM. The same protein was also titrated with NaNO_2_, ammonia and uric acid (Fig. 1B), but an absence of binding was noted in all cases, confirming the thermal shift assay results. We were intrigued by the failure of PA2788-LBD to sense nitrite since other sensor proteins were found to sense nitrate as well as nitrite (34–36). ITC experiments performed with ligands provide information only on higher-affinity binding events. To confirm that PA2788-LBD does not bind nitrite with low affinity, we performed titrations of PA2788-LBD with nitrate in the absence and presence of 20 mM nitrite. In cases of nitrite binding, this would alter nitrate recognition. However, this was not the case (see Fig. S1A in the supplemental material), confirming that PA2788-LBD is a nitrate-specific receptor. Following the demonstration that PA2788 specifically binds nitrate, this chemoreceptor was named McpN.

10.1128/mBio.02334-18.1 FIG S1 Specificity of nitrate recognition at McpN-LBD. (A) Microcalorimetric titration of McpN-LBD with NaNO_3_ in the absence and presence of 20 mM NaNO_2_. (Upper panel) In black, raw data for the titration of 36 µM McpN-LBD with 12.8-μl aliquots of 2 mM NaNO_3_; in red, repetition of the experiment with both ligands containing in addition 20 mM NaNO_2_. (Lower panel) Concentration data representing normalized and dilution heat-corrected integrated peak areas of raw data. The continuous lines represent the curve fits obtained using the “One binding site model” of ORIGIN. (B) Superimposition of the nitrate molecule in the structures of McpN-LBD (in blue) and NarX-LBD (in orange; PDB ID 3EZH). Shown are the amino acid residues in the vicinity of bound nitrate. Hydrogen bonds are indicated by dotted lines. Download FIG S1, TIF file, 2.4 MB. Copyright © 2019 Martín-Mora et al. 2019 Martín-Mora et al. This content is distributed under the terms of the Creative Commons Attribution 4.0 International license.

### Nitrate is not recognized by the LBDs of the PilJ and PA4520 chemoreceptors.

Inspection of the chemoreceptor repertoire of P. aeruginosa (9) suggests that two other chemoreceptors may also bind nitrate. First, the LBD of the PilJ (PA0411) receptor is composed of two consecutive PilJ domains (9). Second, chemoreceptor PA4520 was predicted to contain a NIT LBD (Pfam PF08376). This domain, representing approximately 3% of all extracellular prokaryotic LBDs (21), was predicted to recognize nitrate and nitrite (37).

To verify whether these receptors also bind nitrate, we generated the purified individual LBDs of both receptors for thermal shift assay ligand screening using the PM3B array. In the absence of ligand, PilJ-LBD and PA4520-LBD unfolded with *T_m_* values of 41.8 and 34.4°C, respectively. In both cases, however, no significant ligand-induced *T_m_* shifts were noted (Fig. S2A and B) and microcalorimetric titrations confirmed the absence of NaNO_3_ and NaNO_2_ binding (Fig. S2C). To exclude the possibility that endothermic and exothermic contributions to binding canceled out each other at a given analysis temperature, the experiments were repeated at 15°C; however, the same result was produced, confirming the absence of nitrate/nitrite binding to PilJ-LBD and PA4520-LBD.

10.1128/mBio.02334-18.2 FIG S2 The absence of nitrite and nitrate binding to PilJ-LBD and PA4520-LBD. (A and B) Results from thermal shift assays. Shown are changes in the *T_m_* values of PilJ-LBD (A) and PA4520-LBD (B) in the presence of compounds from Biolog array PM3B. (C) Microcalorimetric titration of PilJ-LBD and PA4520-LBD with NaNO_3_ and NaNO_2_. As a control, the titration of buffer with NaNO_3_ is shown. The injection volume was 19.2 µl. Download FIG S2, TIF file, 2.6 MB. Copyright © 2019 Martín-Mora et al. 2019 Martín-Mora et al. This content is distributed under the terms of the Creative Commons Attribution 4.0 International license.

### McpN mediates nitrate chemotaxis under nitrate starvation conditions.

To assess the function of McpN, we conducted quantitative capillary chemotaxis assays for NaNO_3_ using the wild-type (wt) strain as well as a mutant deficient in the *mcpN* gene. These assays were conducted using the standard conditions that we routinely employ to study P. aeruginosa chemotaxis. This assay involves cell culture in MS minimal medium (4.2 g/liter Na_2_HPO_4_, 2.8 g/liter KH_2_PO_4_, 2.0 g/liter NH_4_NO_3_, 0.2 g/liter MgSO_4_ 7H_2_O, 17.0 mg/liter FeCl_3_ 6H_2_O, 0.8 mg/CoCl_2_ 6H_2_O, 0.6 mg/liter CaCl_2_ 2H_2_O, 0.3 mg/liter Na_2_MoO_4_ 2H_2_O, 0.1 mg/liter H_3_BO_3_, 0.2 mg/liter ZnSO_4_ 7H_2_O, 0.2 mg/liter CuSO_4_ 7H_2_O, 0.2 mg/liter MnSO_4_ 7H_2_O) supplemented with glucose (note that this medium also contains 25 mM NH_4_NO_3_ as a nitrogen source). However, only very minor, nonsignificant responses were detected over the entire nitrate concentration range (Fig. S3).

10.1128/mBio.02334-18.3 FIG S3 Quantitative capillary chemotaxis assays of P. aeruginosa PAO1 and the *mcpN* mutant under conditions of abundant nitrate. Cells were grown in M9 minimal medium supplemented with glucose as a carbon source. This medium contains 25 mM ammonium nitrate as a nitrogen source. Data were corrected using the number of cells that swam into buffer containing capillaries (1,992 ± 298). Download FIG S3, TIF file, 0.07 MB. Copyright © 2019 Martín-Mora et al. 2019 Martín-Mora et al. This content is distributed under the terms of the Creative Commons Attribution 4.0 International license.

**FIG 2:**
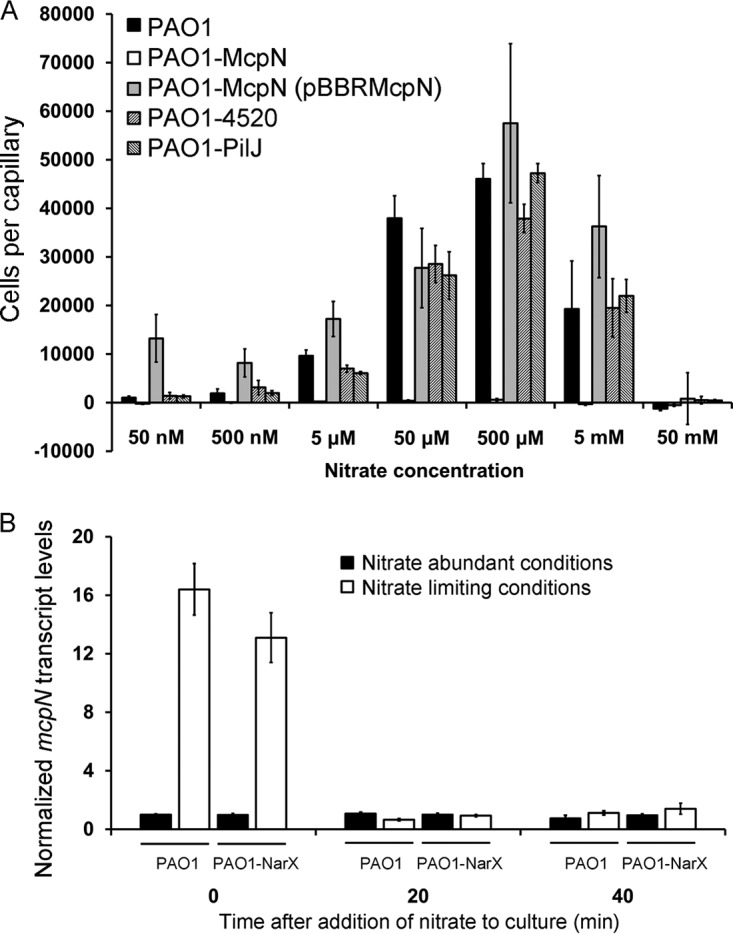
The McpN chemoreceptor of P. aeruginosa mediates nitrate chemotaxis. (A) Quantitative capillary chemotaxis assays of different P. aeruginosa PAO1 strains at different NaNO_3_ concentrations. Cells were grown in rich 2× YT medium and then diluted 133-fold into N0 medium (deficient in nitrogen sources). Data represent means of results from three biological replicates conducted in triplicate. (B) RT-qPCR analysis of the *mcpN* transcript in the wild-type strain and in a mutant defective in the NarX sensor kinase. Cells were gown in MS medium supplemented with glucose (containing 25 mM NH_4_NO_3_) or in nitrate-deficient N0 medium (inoculated using a culture grown in 2× YT medium) until an OD_600_ of 0.15 was reached (time zero), at which point NaNO_3_ was added to reach a final concentration of 1 mM. Further samples were taken after 20 and 40 min. Shown are *mcpN* transcript levels normalized with respect to the transcript levels of the *rpoD* reference gene at time zero under conditions of nitrate abundance. Data represent means and standard deviations of results from three biological replicates conducted in triplicate.

Previous studies have shown that chemotaxis to P_i_ in PAO1 was not observed in rich medium containing significant amounts of P_i_ but was induced by P_i_ starvation (23). We hypothesized that this might also be the case for nitrate chemotaxis and followed an approach similar to that described previously by Wu et al. (23). Thus, cells were precultured in rich 2× YT medium (10 g yeast extract liter^−1^, 16 g Bacto tryptone liter^−1^, 10 g NaCl liter^−1^) and then diluted 133-fold into N0 medium (which lacks nitrogen sources) and continued to grow for another 3 h. Under these conditions, strong chemotactic responses to NaNO_3_ were obtained. Initial significant responses were obtained at a NaNO_3_ concentration of 5 µM, whereas maximal responses were observed at 500 µM (Fig. 2). No nitrate chemotaxis was observed for the *mcpN* mutant, suggesting that it is the sole nitrate chemotaxis receptor. The complementation of this mutant with a plasmid harboring the *mcpN* gene restored nitrate chemotaxis (Fig. 2).

To identify the possible roles of the PilJ receptor and the NIT domain containing PA4520 chemoreceptor in nitrate chemotaxis, single mutants with mutations of the corresponding genes were also analyzed. As shown in Fig. 2A, their responses to nitrate were similar to those seen with the wt strain, confirming that the observed nitrate chemotaxis was mediated solely by McpN.

### Nitrate reduces *mcpN* transcript levels.

To explain the absence of taxis under conditions of nitrate abundance, we hypothesized that nitrate might repress expression of the *mcpN* gene. To verify this hypothesis, we quantified *mcpN* transcript levels by reverse transcription-quantitative PCR (RT-qPCR). These assays were carried out using RNA from cells grown using the same protocol used for the chemotaxis assays under conditions of nitrate abundance and limitation. As shown in Fig. 2B, *mcpN* transcript levels were approximately 16 times higher under nitrate-limiting conditions than under nitrate-abundant conditions. To verify that the absence or presence of nitrate was the cause for these differences, NaNO_3_ was added to these cultures to reach a final concentration of 1 mM and samples were taken for RT-qPCR experiments after additional periods of growth of 20 and 40 min. The results showed that the addition of nitrate to cells grown under nitrate-limiting conditions reduced *mcpN* transcript levels to those seen under nitrate-abundant conditions, indicating that nitrate reduces *mcpN* expression (Fig. 2B).

The NarX/NarL two-component system (TCS) senses nitrate and regulates genes involved in nitrate metabolism (38). To identify a potential role of this TCS in *mcpN* expression, we quantified *mcpN* transcript levels in a mutant defective in the gene encoding the NarX sensor kinase. However, RT-qPCR data revealed no statistical differences in the transcript levels of *mcpN* (Fig. 2B).

### McpN signals through the Che pathway.

The 26 PAO1 chemoreceptors signal through four different chemosensory pathways, and McpN was predicted to signal through the Che pathway (11). To verify this prediction, we conducted chemotaxis assays for NaNO_3_ using mutants defective in the genes encoding the CheA paralogues of the Che1 (CheA_1_) and Che2 (CheA_2_) pathways. As shown in Fig. S4, no nitrate chemotaxis was observed in the *cheA1* mutant, whereas the responses of the *cheA2* mutant were comparable to wt levels. These results thus confirm that McpN signals through the Che pathway (11).

10.1128/mBio.02334-18.4 FIG S4 Quantitative capillary chemotaxis assays of P. aeruginosa PAO1 and mutants deficient in *cheA1* (*PA1458*) and *cheA2* (*PA0178*) in the presence of 500 µM NaNO_3_. Data represent means and standard deviations of results from three biological replicates conducted in triplicate. Download FIG S4, TIF file, 1.4 MB. Copyright © 2019 Martín-Mora et al. 2019 Martín-Mora et al. This content is distributed under the terms of the Creative Commons Attribution 4.0 International license.

### The three-dimensional (3D) structure of McpN-LBD.

**FIG 3:**
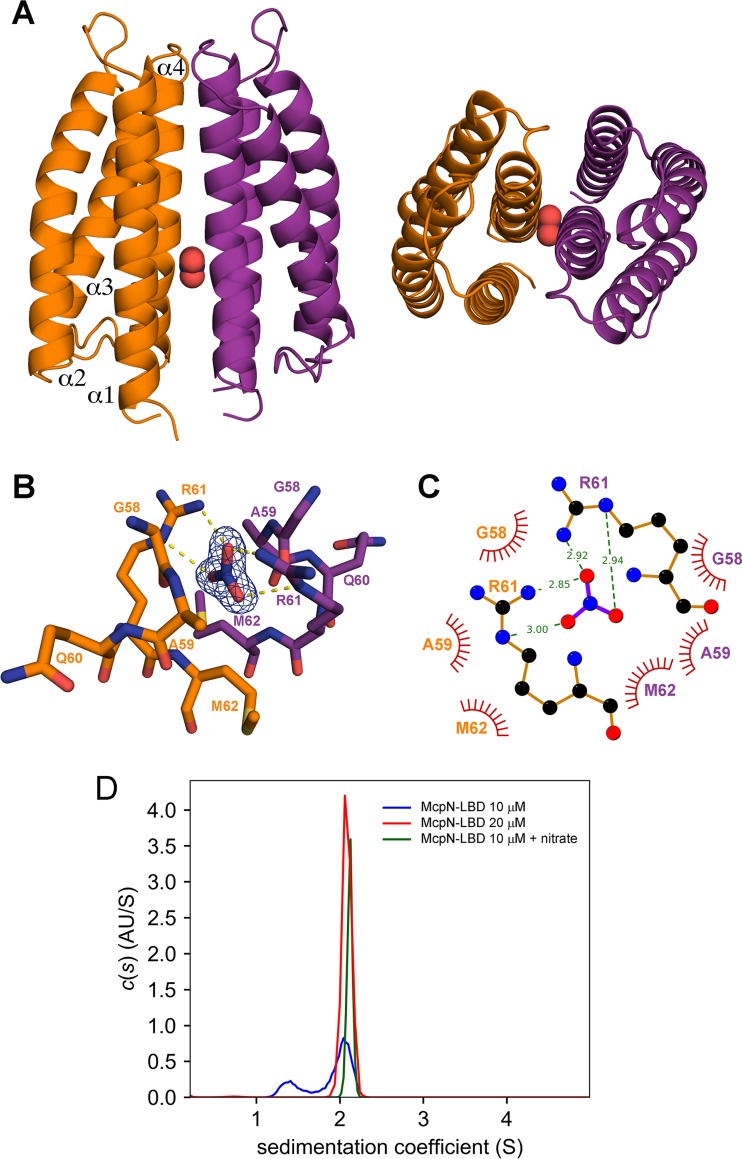
The three-dimensional structure of McpN-LBD in complex with nitrate. (A) Side (left) and top (right) views of the overall structure. Nitrate is shown in red. (B) The nitrate binding pocket. Shown are amino acids from both chains that interact with nitrate. The |2Fo-Fc| electron density of nitrate is contoured at 1.5 sigma. (C) Schematic representation of amino acids involved in hydrogen bonding to nitrate, shown as dotted lines, while the spoked arcs represent residues that make nonbonded contacts. (D) Analysis of the oligomeric state of McpN-LBD using sedimentation velocity analytical ultracentrifugation (AU). Shown are the sedimentation velocity *c*(*s*) profiles of ligand-free McpN-LBD at different concentrations and protein in complex with nitrate.

McpN-LBD in complex with nitrate was crystallized in a buffer at pH 7.5, and its structure was resolved by X-ray crystallography to a resolution of 1.3 Å. According to the Matthews coefficient, the unit cell accommodates three chains. A structural alignment of these three chains resulted in root mean square deviation (RMSD) values below 0.5, indicating that these chains can be considered identical. Chains A and B of the unit cell form a dimer (Fig. 3A), whereas chain C forms another dimer with a symmetry-related chain. The McpN-LBD monomer is composed of 4 α-helices that pack into a 4-helix bundle. Dimerization is achieved through the interaction of 22 residues of chains A and B that establish 16 hydrogen bonds and occlude a surface of approximately 1,100 Å^2^ in each monomer.

A single molecule of nitrate is bound to a site with a positive surface charge at the dimer interface. The binding site is situated on the dimer symmetr*y* axis; consequently, the same amino acids from the two monomers establish interactions with bound nitrate. Whereas G58, A59, and M62 establish nonbonded contacts, R61 played a key role in recognition since it forms two hydrogen bonds with nitrate (Fig. 3B). To verify the role of R61, we generated a McpN-LBD R61A mutant. The intrinsic tryptophan fluorescence emission spectrum (Fig. S5A) and the thermal unfolding properties of the mutant protein (Fig. 1; see also Fig. S5B) were comparable to those seen with the native protein, indicating that this amino acid replacement did not cause major changes to the overall protein structure. Analysis of this protein by the thermal shift assay and ITC showed that this protein was unable to recognize nitrate (Fig. S5B and C).

10.1128/mBio.02334-18.5 FIG S5 Analysis of the McpN-LBD R61A mutant. (A) Intrinsic tryptophan fluorescence emission spectra of McpN-LBD and McpN-LBD R61A. (B) Thermal shift assay of McpN-LBD R61A in the absence and presence of NaNO_3_. (C) Microcalorimetric titration of McpN-LBD R61A with NaNO_3_. Download FIG S5, TIF file, 2.9 MB. Copyright © 2019 Martín-Mora et al. 2019 Martín-Mora et al. This content is distributed under the terms of the Creative Commons Attribution 4.0 International license.

**FIG 4:**
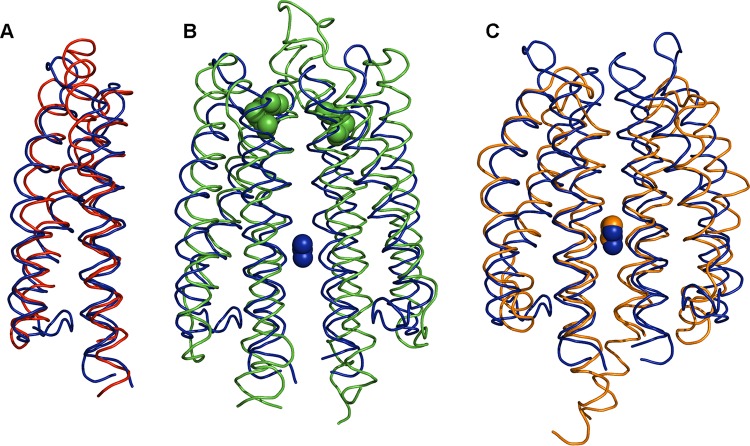
Structural alignment of the McpN-LBD C_∝_ chain with structural homologues. In all cases, McpN-LBD is shown in blue. (A) Alignment with a CHASE3 domain of an uncharacterized histidine kinase of Rhodopseudomonas palustris (PDB ID 3VA9), the closest structural homologue found in a DALI search (Table 1). (B) Alignment with Tar-LBD (PDB ID 1VLT). Bound aspartate (Tar) is shown in green, whereas bound nitrate (McpN) is shown in blue. (C) Alignment with the sensor domain of the NarX histidine kinase (PDB ID 3EZH). Bound nitrates overlap and are shown in blue (McpN) and orange (NarX).

The McpN-LBD structure was aligned to all structures currently deposited in the protein data bank using the DALI algorithm, and the closest structural homologues are listed in Table 1. Surprisingly, the closest structure was a LBD of a histidine kinase that belonged to a different family, namely, CHASE3. The high level of structural similarity between this domain and McpN-LBD is illustrated in Fig. 4A. The only chemoreceptor LBD with significant structural similarity was the HBM domain of the McpS chemoreceptor, which is composed of two 4-helix bundles (39).

**TABLE 1:** Structural alignment of McpN-LBD with structures deposited in the Protein Data Bank^*a*^

PDB ID	Protein type	Species	Ligand	Pfam/InterPro ID	Z- score	No. of aligned residues	Sequence identity (%)	Reference
3VA9	LBD of HK9 SK	Rhodopseudomonas palustris		CHASE3 (PF05227)	13.6	114	13	Unpublished
5XSJ	LBD of LytS SK	Clostridium beijerinckii	XylFII ligand binding protein	Unannotated	13.3	112	6	82
4K0D	LBD of Adeh_2942 SK	Anaeromyxobacter dehalogenans		Unannotated	12.3	117	17	83
2YFB	LBD of McpS CR1	Pseudomonas putida	TCA cycle intermediates	HBM (PF16591)	12.0	113	12	39
3EZH	LBD of NarX SK	Escherichia coli	Nitrate/nitrite	PilJ (PF13675)	11.9	104	21	72
3O1J	LBD of TorS SK	Vibrio parahaemolyticus	TorT periplasmic binding protein	TorS-like (IPR038188)	11.2	111	14	84
4IGG	α-Catenin	Homo sapiens	β-Catenin	Vinculin (PF01044)	10.7	114	5	85
5JEQ	LBD of NarQ SK	Escherichia coli	Nitrate/nitrite	PilJ (PF13675)	10.6	103	15	73
5XA5	α-Catenin	Caenorhabditis elegans	β-Catenin	Vinculin (PF01044)	10.4	110	7	85
5XFL	α-Catenin	Mus musculus	β-Catenin	Vinculin (PF01044)	10.3	114	5	85

a Shown are the structures with a Z-score above 10. The listed structures share less than 90% sequence similarity. SK, sensor kinase; CR, chemoreceptor.

### Nitrate binding promotes McpN-LBD dimerization.

Analytical ultracentrifugation (AUC) experiments were conducted to assess the oligomeric state of McpN-LBD. Initially, we used the three-dimensional structure of McpN-LBD to calculate the expected sedimentation (S) coefficients using HYDROPRO (40) software. This analysis resulted in *s_20_*_,_*_w_* values of 1.7 S and 2.8 S (at 20 degrees and using water) for the monomeric and dimeric species, respectively.

Sedimentation velocity experiments were performed on 5 µM to 40 µM McpN-LBD in the absence of ligand, and data obtained with 10 and 20 µM protein are shown in Fig. 3D. At 10 µM, two peaks could be identified with *s_20_*_,_*_w_* values of 1.85 S and 2.69 S, which fit well with the values determined for the monomer and dimer, respectively. At 20 µM and 40 µM, only protein dimers were observed, indicating that the equilibrium had shifted completely to this oligomeric state. To assess the effect of nitrate binding on the oligomeric state, the experiments described above were repeated in the presence of saturating nitrate concentrations. No changes in oligomeric state occurred at the 20 and 40 µM concentrations, whereas a single peak with *s_20_*_,_*_w_* = 2.79 S (Fig. 3D) was obtained at 10 µM protein, indicating that nitrate binding had shifted the equilibrium entirely to the dimeric state. Taken together, the data indicate that McpN-LBD was present in a monomer-dimer equilibrium and that nitrate binding stabilized the dimeric state.

### Definition of the N-box.

To identify potential McpN homologues in other species, we conducted a BLAST-P search of McpN-LBD in the NCBI database of nonredundant protein sequences, excluding members of the *Pseudomonas* genus. An alignment of the top 87 sequences is shown in Fig. S6. All sequences belonged to the PilJ family and formed part of chemoreceptors. Most of the corresponding species were marine bacteria, and a significant proportion of them are able to oxidize elemental sulfur or sulfite (see Table S1 in the supplemental material). Furthermore, a number of human pathogens such as Enterobacter cloacae, Streptococcus pneumoniae, and Eggerthia catenaformis were among the species that harbor McpN homologues (Table S1).

10.1128/mBio.02334-18.6 FIG S6 Alignment of the McpN-LBD sequences with homologues from other species. The alignment is based on a BLAST-P search of McpN-LBD in the NCBI database of nonredundant sequences, excluding species of the genus *Pseudomonas*. The alignment was done using the CLUSTALW algorithm of npsa software (C. Combet, C. Blanchet, C. Geourjon, and G. Deléage, Trends Biochem Sci 25:147–150, 2000, https://www.cell.com/trends/biochemical-sciences/fulltext/S0968-0004(99)01540-6?_returnURL=https%3A%2F%2Flinkinghub.elsevier.com%2Fretrieve%2Fpii%2FS0968000499015406%3Fshowall%3Dtrue). The BLOSUM62 matrix was used, and the settings used for analysis of gap costs were defined as follows: existence, 11; extension, 1. The McpU-LBD sequence is shaded in yellow. Download FIG S6, PDF file, 0.02 MB. Copyright © 2019 Martín-Mora et al. 2019 Martín-Mora et al. This content is distributed under the terms of the Creative Commons Attribution 4.0 International license.

10.1128/mBio.02334-18.8 TABLE S1 Characteristics of species that contain McpN homologues. A BLAST-P search was conducted in the NCBI database of nonredundant protein sequences (excluding species of the genus *Pseudomonas*), and the top 87 sequences are listed in Fig. S6. This table shows the characteristics of the corresponding bacterial species. Human pathogens are highlighted in green, and bacteria that oxidize sulfurous compounds are highlighted in yellow. Download Table S1, DOCX file, 0.09 MB. Copyright © 2019 Martín-Mora et al. 2019 Martín-Mora et al. This content is distributed under the terms of the Creative Commons Attribution 4.0 International license.

**FIG 5:**
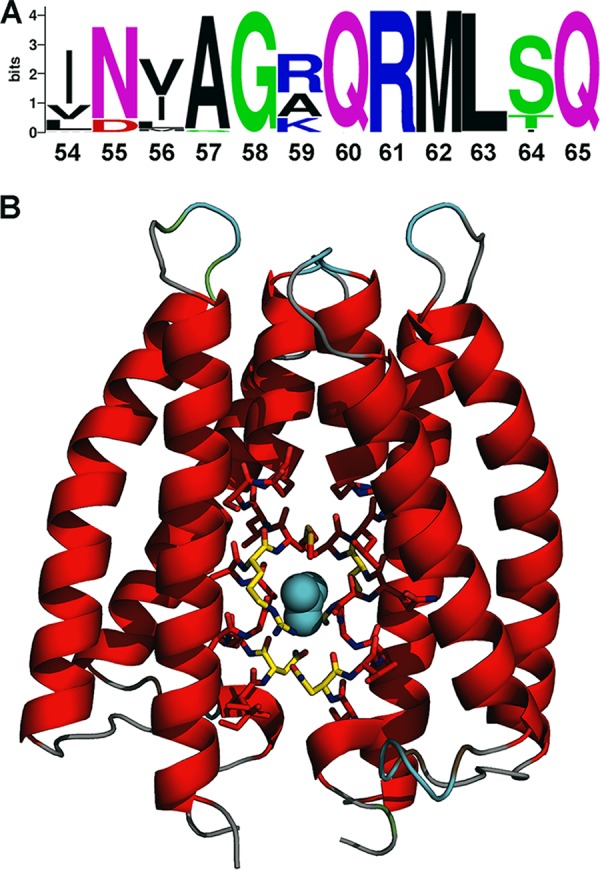
Definition of the N-box of PilJ domains. (A) The sequence logo of the N-box as derived from the alignment shown in Fig. S6. (B) Structure of McpN-LBD in which the 12 amino acids of the N-box are shown as sticks together with bound nitrate.

The sequence alignment of McpN-LBD homologues revealed only a very modest level of overall sequence identity of approximately 5%. However, the zone around the nitrate binding site, which we have termed the N-box, was highly conserved and the corresponding sequence logo is shown in Fig. 5A. We have shown above that not all PilJ domains bind nitrate, since no binding was observed for the PilJ LBD, which is composed of two PilJ domains (Fig. S2). As shown in Fig. S7, the N-box was not conserved in either of the PilJ domains of the PilJ chemoreceptor. We then scanned the TrEMBL database using PROSITE (41) and the following consensus pattern for the N-box: [IVL]-[ND]-x-A-G-x-Q-R-M-L-[ST]-Q. The random statistical probability of a match was well below 1 sequence.

10.1128/mBio.02334-18.7 FIG S7 Sequence alignment of the PilJ domains of the McpN and PilJ chemoreceptors of P. aeruginosa PAO1. PilJ-pilJ1 corresponds to the N-terminal PilJ domain (amino acids 36 to 163), which is followed in sequence by the PilJ-pilJ2 domain (amino acids 164 to 314). The N-box in McpN is shaded in yellow. Red, identical residues; green, highly similar residues; blue, weakly similar residues. The alignment was done using the CLUSTALW algorithm of npsa software (Combet et al., 2000). The BLOSUM62 matrix was used, and the settings used for analysis of gap costs were defined as follows: existence, 11; extension, 1. Download FIG S7, TIF file, 0.6 MB. Copyright © 2019 Martín-Mora et al. 2019 Martín-Mora et al. This content is distributed under the terms of the Creative Commons Attribution 4.0 International license.

However, 941 sequences containing PilJ domains which are likely to be nitrate binding domains were retrieved. The retrieved sequences formed part of all major families of signal transduction systems, namely, transcriptional regulators, sensor kinases, chemoreceptors, and diguanylate cyclases. There were 1,135 protein sequences with at least one PilJ domain in Pfam at the time of the search, and the N-box may be usable as a means to identify PilJ domains that are able to bind nitrate.

### Nitrate chemotaxis in other bacterial species.

**FIG 6:**
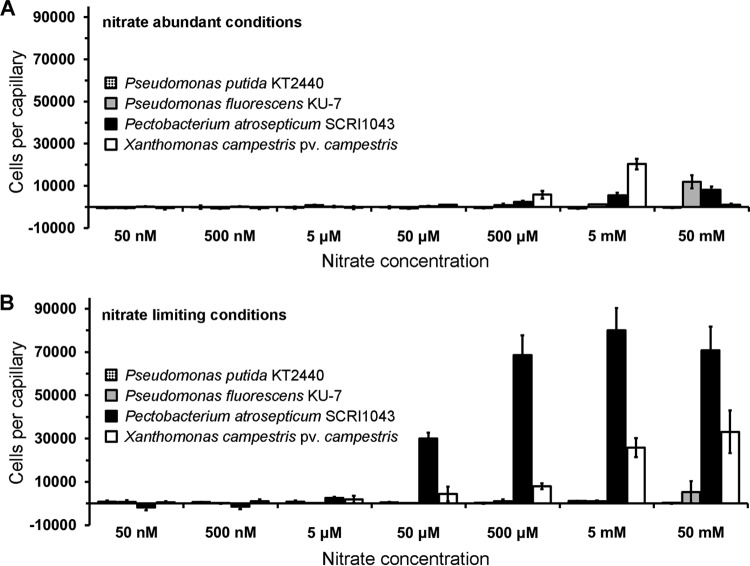
Nitrate chemotaxis in other species. Quantitative capillary chemotaxis assays of different strains to NaNO_3_. (A) Cells grown under conditions of nitrate abundance. (B) Cells grown under conditions of nitrate limitation. Data represent means and standard deviations of results from three biological replicates conducted in triplicate.

Subsequent work was aimed at assessing nitrate chemotaxis in other species. To that end, we conducted quantitative capillary chemotaxis assays using different strains grown under conditions of nitrate abundance and limitation. Pseudomonas putida KT2440 and Pseudomonas fluorescens KU-7 do not have an McpN homologue but contain, as in the case of P. aeruginosa, a NIT domain containing chemoreceptor. Our experiments showed that P. putida KT2440 was devoid of nitrate chemotaxis whereas P. fluorescens showed only minor responses to 50 mM nitrate (Fig. 6).

We then studied two bacterial species that are among the top 10 plant-pathogenic bacteria (42), namely, Pectobacterium atrosepticum and Xanthomonas campestris pv. *campestris.* Interestingly, the responses of P. atrosepticum were very similar to those of PAO1, since only very minor responses were observed under nitrate abundance conditions but strong responses were observed under nitrate starvation conditions (Fig. 6). There is no McpN homologue among the 36 chemoreceptors of this strain, but there is a single receptor with a NIT domain. X. campestris pv. *campestris* also showed significant chemotaxis under nitrate-limiting conditions and only minor responses under nitrate abundance conditions (Fig. 6). Altogether, these data suggest that the induction of nitrate chemotaxis by nitrate limitation is common to other bacteria.

## DISCUSSION

Nitrate is a final electron acceptor for anaerobic respiration and also serves as a nitrogen source for aerobic growth. Taxis to nitrate has been observed for a significant number of bacteria such as E. coli, Salmonella enterica serovar Typhimurium (43, 44), *Pseudomonas* spp. (45–48), *Shewanella* spp. (49, 50), Azospirillum brasilense (51), Rhodobacter sphaeroides, Agrobacterium tumefaciens (52), *Thioploca* spp. (53), and *Synechococcus* spp. (54). The three major bacterial pathways for nitrate metabolism include respiratory, assimilatory, and dissimilatory nitrate reduction (55), and any type of metabolism can lead to energy taxis. In some of the cases, it has been demonstrated that bacterial nitrate taxis is based on energy taxis (44, 49, 51, 52). For example, deletion or inhibition of enzymes that participate in nitrate metabolism abolished nitrate taxis (49). In other reports, the molecular mechanism of nitrate taxis, such as in the case of, for example, lake water bacteria (46) or different denitrifying strains (45, 47), is unclear. It is possible that those observations were based on chemotaxis. Here we identify the molecular mechanism of nitrate-specific chemotaxis that is initiated by the specific recognition of nitrate at a periplasmic chemoreceptor LBD. McpN homologues show a broad phylogenetic distribution, including those of archaea and bacteria belonging to the *Firmicutes* and *Proteobacteria* phyla (see Table S1 in the supplemental material), which indicates that nitrate chemotaxis may be a widespread mechanism.

Interestingly, among the species that harbor McpN homologues were a significant number of bacteria isolated from marine sediments that are able to oxidize sulfide or elemental sulfur (Table S1). There is evidence that the oxidation of reduced sulfur compounds in these bacteria is coupled to the reduction of electron acceptors such as nitrate (56). As a consequence, some sulfide oxidizers were found to store nitrate in vacuoles (57) at concentrations of up to 370 mM (58, 59). This intracellular nitrate is used to oxidize sulfide in deeper anoxic zones of sediments. This process has been particularly well studied in *Beggiatoa* spp. (60), which are also among the species that contain an McpN homologue (Table S1). On the basis of experiments performed with nitrate-reducing/sulfide-oxidizing shelf sediment bacteria belonging to the *Thioploca* genus, a functional model was proposed (53). The authors showed that the nitrate concentration in the sediment was lower than that in the flume water and that nitrate chemotaxis directed bacteria to the sediment surface, where they filled their vacuoles with nitrate. They then migrated back into deeper sediment layers, where they oxidized sulfide to sulfate until the nitrate was depleted, which induced the upward movement. Taken together, the data thus suggest the particular importance of nitrate chemotaxis in marine sulfide/sulfur-oxidizing bacteria.

Nitrate serves PAO1 as the sole nitrogen source for growth, and the anaerobic growth of this strain is accomplished through the denitrification enzyme pathway that catalyzes the sequential reduction of nitrate to nitrogen gas (61). Nitrate chemotaxis was observed in pathogenic P. aeruginosa bacteria but not in the nonpathogenic P. putida and P. fluorescens, suggesting that it may be related to virulence. Previous studies have shown a link between virulence and nitrate metabolism for anaerobically grown PAO1, since a mutant with a mutation in the nitrate reductase gene was avirulent in Caenorhabditis elegans (61). PAO1 causes airway infections in cystic fibrosis patients, and the sputum nitrate/nitrite concentration was 774 µM in cystic fibrosis patients, well above the concentration seen with the healthy control group (421 µM) (62). Importantly, these concentrations are in the range of the optimal chemotaxis responses measured here (Fig. 2), indicating that nitrate chemotaxis may be related to pathogenicity, as in the case of *S.* Typhimurium, where taxis to host-derived nitrate is required for efficient host infection (44).

PAO1 nitrate chemotaxis was observed only under nitrate starvation conditions (Fig. 2A), whereas no taxis was observed in under nitrate abundance conditions (see Fig. S3 in the supplemental material), and similar results were seen for P. atrosepticum (Fig. 6). This is unusual, since chemotactic behaviors are typically either constitutive or inducible by the chemoeffector (63, 64). However, striking similarities exist between P_i_ and nitrate chemotaxis in PAO1. P_i_ taxis was observed only under P_i_ starvation conditions and not under P_i_ abundance conditions (23, 24). As in the case of *mcpN*, the presence of P_i_ was shown to decrease the transcript levels of both P_i_ chemoreceptor genes, i.e., *ctpL* and *ctpH* (65). P_i_ was identified as a key signal molecule that controls the expression of many virulence genes and features in PAO1 (65, 66). P_i_ and nitrate are both inorganic anions, and it is tempting to speculate that chemotaxis repression mediated by chemoeffector abundance is a feature of this compound family.

Almost one-third of all chemoreceptor LBDs are recognized by the Pfam 4HB domain signature (9). Signaling of chemoreceptors with this domain has been extensively studied, and the 3D structure reveals a 4-helix antiparallel bundle (67, 68). Although the McpN-LBD sequence is not recognized by the Pfam 4HB signature, its structure superimposes very well on that of the 4HB Tar-LBD (Fig. 4B). This, together with the fact that the closest structural McpN-LBD homologue is a CHASE3 domain (Table 1), demonstrates that the 4-helix bundle is a conserved structural motif for ligand sensing formed by members of different LBD families. Although conserved in structure, the modes of ligand binding for McpN-LBD and Tar-LBD are different. The Tar-LBD dimer recognizes two signal molecules with high negative cooperativity that bind to the dimer interface at two sites that are not on the dimer symmetr*y* axis (68, 69). In contrast, a single molecule of nitrate binds to a single site located at the dimer symmetr*y* axis of McpN-LBD (Fig. 3A). However, 4HB domains and McpN-LBD (Fig. 3D) have in common that the individual domains are present in a monomer-dimer equilibrium and that ligand binding shifts this equilibrium to the dimeric state (69, 70).

The NarX/NarL and NarQ/NarP two-component systems control transcriptional responses to nitrate and nitrite, which are the preferred anaerobic electron acceptors in E. coli (71). The LBDs of the NarX and NarQ sensor kinases are structural homologs of McpN-LBD (Table 1), and their 3D structures in complex with nitrate have been solved (72, 73). Although McpN-LBD and NarX-LBD share only 21% sequence identity, their structures align very well and the nitrate binding site is conserved (Fig. 4C). McpN-LBD differs from NarX-LBD in several aspects. Our AUC studies showed that McpN-LBD has an intrinsic propensity to dimerize which is enhanced in the presence of nitrate. In contrast, NarX-LBD is monomeric even at a concentration of 10 mM and in the presence of nitrate (72). NarX and NarQ are characterized by a certain plasticity in ligand recognition, since they bind to nitrate, nitrite, and sulfite (34–36). In contrast, McpN-LBD recognizes nitrate exclusively and has no physiologically relevant affinity for nitrite (Fig. 1; see also Fig. S1A). The superimposition of the ligand binding pockets of NarX-LBD and McpN-LBD (Fig. S1B) did not provide any obvious reason for this difference in ligand specificity data.

The NIT domain is present in different signal transduction protein families and was previously proposed to be a sensor domain for nitrate and nitrite (37). However, the recombinant NIT domain of PA4520 did not bind nitrate or nitrite (Fig. S2) and a mutant defective in this receptor was not affected in nitrate chemotaxis (Fig. 2). In addition, P. putida and P. fluorescens both possess a NIT domain containing a chemoreceptor which, however, did not mediate nitrate chemotaxis under the experimental conditions tested (Fig. 6). The NIT domain may thus represent a superfamily that contains subfamilies with different ligand binding properties and biological functions.

The demonstration of specific nitrate chemotaxis as reported here widens the range of known chemoeffectors and provides the basis for an assessment of this phenotype in other bacteria and for the elucidation of its physiological relevance.

## MATERIALS AND METHODS

### Bacterial strains, culture media, and growth conditions.

Bacterial strains used are listed in Table 2. Bacteria were grown aerobically at 30°C or 37°C, unless otherwise specified, in lysogeny broth (LB), 2× YT medium (10 g yeast extract liter^−1^, 16 g Bacto tryptone liter^−1^, 10 g NaCl liter^−1^), or MS medium (4.2 g/liter Na_2_HPO_4_, 2.8 g/liter KH_2_PO_4_, 2.0 g/liter NH_4_NO_3_, 0.2 g/liter MgSO_4_ 7H_2_O, 17.0 mg/liter FeCl_3_ 6H_2_O, 0.8 mg/CoCl_2_ 6H_2_O, 0.6 mg/liter CaCl_2_ 2H_2_O, 0.3 mg/liter Na_2_MoO_4_ 2H_2_O, 0.1 mg/liter H_3_BO_3_, 0.2 mg/liter ZnSO_4_ 7H_2_O, 0.2 mg/liter CuSO_4_ 7H_2_O, 0.2 mg/liter MnSO_4_ 7H_2_O) supplemented with 20 mM d-glucose as a carbon source. Alternatively, Xanthomonas campestris was grown in M9 minimal medium supplemented with 20 mM d-glucose, 5 mM NaNO_3_ and 5% (vol/vol) LB medium. E. coli DH5α was used as a host for gene cloning. When necessary, antibiotics were used at the following final concentrations (in micrograms per milliliter): ampicillin, 100; kanamycin, 50, tetracycline, 40.

**TABLE 2:** Bacterial strains and plasmids used in this study

Strain or plasmid	Genotype or relevant characteristic(s)^*a*^	Reference or source
Strains		
Escherichia coli BL21(DE3)	F^–^ *ompT gal dcm lon hsdS*_B_(r_B_^–^ m_B_^–^) λ(DE3 [*lacI lacUV5*-*T7p07 ind1 sam7 nin5*]) [*malB*^+^]_K-12_(λ^S^)	86
Escherichia coli DH5α	F^–^ *endA1 glnV44 thi-1 recA1 relA1 gyrA96 deoR nupG purB20* φ80d*lacZ*ΔM15 Δ(*lacZYA-argF*)*U169*, *hsdR17*(r_K_^–^ m_K_^+^), λ^–^	87
Pseudomonas putida KT2440	Wild-type strain	88
Pseudomonas aeruginosa PAO1	Wild-type strain	89
Pseudomonas aeruginosa PAO1-McpN	PAO1 transposon mutant *pa2788*::IS*lacZ*/hah; Tc^r^	90
Pseudomonas aeruginosa PAO1-PilJ	PAO1 transposon mutant *pa0411*::IS*phoA*/hah; Tc^r^	90
Pseudomonas aeruginosa PAO1-PA4520	PAO1 transposon mutant *pa4520*::IS*phoA*/hah; Tc^r^	90
Pseudomonas aeruginosa PAO1-NarX	PAO1 transposon mutant *pa*3878::IS*phoA*/hah; Tc^r^	90
Pseudomonas aeruginosa PAO1*-*CheA1	PAO1 transposon mutant *pa1458*::IS*phoA*/hah; Tc^r^	90
Pseudomonas aeruginosa PAO1*-*CheA2	PAO1 transposon mutant *pa0178*::IS*lacZ*/hah; Tc^r^	90
Pseudomonas fluorescens KU-7	Wild-type strain	91
Pectobacterium atrosepticum SCRI1043	Wild-type strain	92
Xanthomonas campestris pv. *campestris*	Wild-type strain	M. Milagros- Lopez (IVIA, Spain)

Plasmids		
pET28b(+)	Protein expression plasmid; Km^r^	Novagen
pMcpN-LBD	pET28b(+) derivative containing a DNA fragment encoding McpN-LBD cloned into the NdeI/XhoI sites; N*-*terminal His6 tag; Km^r^	This study
pPilJ-LBD	pET28b(+) derivative containing a DNA fragment encoding PilJ-LBD cloned into the NdeI/EcoRI sites; N*-*terminal His6 tag; Km^r^	This study
pET4520-LBD	pET28b(+) derivative containing a DNA fragment encoding PA4520-LBD cloned into the NdeI/SalI sites; N*-*terminal His6 tag; Km^r^	This study
pBBR1MCS2_START	*oriRK2 mobRK2*; Km^r^	93
pBBRMcpN	pBBR1MCS2_START derivative containing *mcpN* gene; Km^r^	This study
pCR2.1-TOPO	PCR cloning vector; *ori* pUC *ori* f1 *lacZα*; Ap^r^, Km^r^	Invitrogen
pCR-McpN-LBD	pTOPO derivative containing a DNA fragment encoding McpN-LBD; Ap^r^, Km^r^	This study
pCR-McpN-R61A	pTOPO derivative containing a DNA fragment encoding McpN-LBD (*R61A*); Ap^r^, Km^r^	This study
pMcpN-R61A	pET28b derivative containing a DNA fragment encoding His-tagged McpN-LBD (*R61A*); Km^r^	This study

a Ap, ampicillin; Km, kanamycin; Tc, tetracycline.

### Plasmid construction.

The plasmids and oligonucleotides used are listed in Table 2 and in Table S2 in the supplemental material, respectively. Protein expression plasmids were constructed by amplification from genomic DNA of P. aeruginosa PAO1 for the DNA fragments encoding the LBDs of PilJ (amino acids 36 to 315), McpN (amino acids 44 to 179), and PA4520 (amino acids 38 to 321). The resulting PCR products were cloned into pET28(+) to generate plasmids pPilJ-LBD, pMcpN-LBD, and pET4520-LBD. In all cases, plasmids were verified by sequencing. For the construction of the complementing plasmid pBBRMcpN, the *mcpN* gene was amplified using primers listed in Table S2. The resulting PCR fragment was cloned into the NdeI and BamHI sites of pBBR1MCS2_START, and the plasmid was transformed into P. aeruginosa PAO1-McpN by electroporation.

10.1128/mBio.02334-18.9 TABLE S2 Oligonucleotides used in this study. Download Table S2, DOCX file, 0.02 MB. Copyright © 2019 Martín-Mora et al. 2019 Martín-Mora et al. This content is distributed under the terms of the Creative Commons Attribution 4.0 International license.

### Protein overexpression and purification.

E. coli BL21(DE3) was transformed with the expression plasmids, and the resulting strains were grown in 2-liter Erlenmeyer flasks containing 400 ml LB medium supplemented with kanamycin. Cultures were grown under conditions of continuous stirring (200 rpm) at 30°C. The growth temperature was lowered to 16°C when an optical density at 600 nm (OD_600_) of 0.5 was reached, and protein expression was induced after 30 min by the addition of 0.1 mM isopropyl β-d-1-thiogalactopyranoside. Cultures were grown for another 14 h prior to harvesting of cells by centrifugation at 10,000 × *g* and 4°C for 30 min. Cell pellets were resuspended in buffer A (20 mM Tris-HCl, 0.1 mM EDTA, 300 mM NaCl, 10 mM imidazole, 5% [vol/vol] glycerol, pH 7.6) and broken by French press treatment at a gauge pressure of 62.5 lb/in^2^. After centrifugation at 20,000 × *g* for 1 h, the supernatant was loaded onto a 5-ml HisTrap column (Amersham Bioscience) previously equilibrated with buffer A. After washing with buffer A containing 35 mM imidazole was performed, protein was eluted by the use of a 35 to 500 mM imidazole gradient in buffer A. Proteins were dialyzed into the following buffers for analysis: for PA2788-LBD, 20 mM Tris-HCl (pH 7.4); for PA4520-LBD, 5 mM Tris-HCl, 5 mM MES (morpholineethanesulfonic acid), and 5 mM PIPES [piperazine-*N*,*N*′-bis(2-ethanesulfonic acid)] (pH 7.5); for PA0411-LBD, 50 mM HEPES (pH 7.5).

### Differential scanning fluorimetry (DSF).

DSF assays were performed on a MyIQ2 Real-Time PCR instrument (Bio-Rad). Compounds from different arrays (Biolog, Hayward, CA, USA) were dissolved in 50 μl water, which, according to the manufacturer, corresponds to a concentration of 10 to 20 mM. The composition of these arrays is provided in http://208.106.130.253/pdf/pm_lit/PM1-PM10.pdf. Screening was performed using 96-well plates. Each well contained 2.5 μl of the dissolved compound, 20.5 μl protein, and 2 μl SYPRO Orange (Life Technologies). The control well contained protein without ligand. Samples were heated from 23°C to 85°C at a scan rate of 1°C/min, and fluorescence changes were monitored. *T_m_* values correspond to the minima of the first derivatives of the raw data.

### Isothermal titration calorimetry (ITC).

Experiments were performed on a VP microcalorimeter (Microcal, Amherst, MA, USA) at 25°C. Proteins were placed into the sample cell (36 to 65 µM). Compound solutions (1 to 5 mM) were prepared in dialysis buffer and placed into the injector syringe. Titrations involved the injection of 9.6-µl to 19.2-µl aliquots of compound solution into the protein. In cases in which no binding was observed, the experiment was repeated at an analysis temperature of 15°C. The mean enthalpy values from the titration of buffer with compounds were subtracted from raw titration data prior to data analysis performed with the “One binding site model” of the MicroCal version of ORIGIN.

### Analytical ultracentrifugation (AUC).

Experiments were performed on a Beckman Coulter Optima XL-I analytical ultracentrifuge (Beckman-Coulter, Palo Alto, CA, USA) equipped with UV-visible light absorbance and interference optics detection systems, using an An50Ti 8-hole rotor and 12-mm-path-length charcoal-filled epon double-sector centerpieces. The experiments were carried out at 10°C using 5 μM to 40 μM McpN-LBD in the absence and presence of 0.6 mM NaNO_3_.

Sedimentation velocity (SV) runs were carried out at a rotor speed of 48,000 rpm using 400-µl samples with the dialysis buffer as the reference. A laser was used at a wavelength of 235 nm in the absorbance optics mode. Least-squares boundary modeling of the SV data was used to calculate sedimentation coefficient distributions with the size-distribution *c*(*s*) method (74) implemented in SEDFIT v14.1 software. Buffer density (*ρ* = 1.003 g/ml [0.99989 g/ml in the presence of NaNO_3_]) and viscosity [*η* = 0.013137 poise [0.01313 poise in the presence of NaNO_3_]) at 10°C were estimated using SEDNTERP software (75) for the buffer components. The partial specific volume used was 0.7192 ml/g as calculated from the amino acid sequence using SEDNTERP software.

### Intrinsic tryptophan fluorescence spectroscopy.

McpN-LBD and McpN-LBD R61A mutants were dialyzed into 20 mM Tris-HCl (pH 7.4), and the reaction mixtures were adjusted to a concentration of 5 μM. Proteins were placed into a PTI QM-2003 fluorimeter (Photon Technology International, Lawrenceville, NJ), and emission spectra were recorded at wavelengths of 305 to 400 nm following excitation at 295 nm. Spectra were recorded at 20°C using a slit width of 4 nm with a scan speed of 1 nm/s. Spectra were corrected with the buffer emission spectrum.

### Quantitative capillary chemotaxis assays.

Assays were conducted using two different protocols that differed under the cell culture conditions. Under conditions of nitrate abundance, overnight cultures in MS minimal medium supplemented with 20 mM glucose as a carbon source (note that this medium contains 25 mM NH_4_NO_3_) were used to inoculate fresh medium to reach an OD_600_ of 0.05. Cells were cultured at 30°C or 37°C until an OD_600_ of 0.4 to 0.5 was reached. Under conditions of nitrate limitation, 150 µl of an overnight culture in rich 2× YT medium was used to inoculate 20 ml of N0 medium (MS lacking a nitrogen source). Growth was continued for 3 h (pseudomonads) or 4.5 h (P. atrosepticum), at which point the cells had reached an OD_600_ of 0.15 to 0.2. For X. campestris, M9 minimal medium supplemented with 20 mM d-glucose, 5 mM NaNO_3_, and 5% (vol/vol) LB was used for the conditions of nitrate abundance, whereas M8 minimal medium (M9 without nitrogen source) supplemented with 20 mM d-glucose and 5% (vol/vol) LB was used for the conditions of nitrate limitation. Cells were grown for 6 h until the OD_600_ reached 0.25 to 0.3.

Under both conditions, cells were washed twice by centrifugation (1,667 × *g* and 6 min at 4°C) and resuspension in chemotaxis buffer (50 mM potassium phosphate, 20 µM EDTA, 0.05% [vol/vol] glycerol, pH 7.0) and then resuspended in the same buffer to reach an OD_600_ of 0.1. Aliquots (230 µl) of the resulting cell suspension were placed into the wells of a 96-well microtiter plate. Capillaries (Microcaps; Drummond Scientific [reference P1424]) (1 μl) were heat-sealed at one end and filled with buffer (control) or chemoeffector solution prepared in chemotaxis buffer. The capillaries were immersed into the bacterial suspensions at its open end. After 30 min, capillaries were removed from the wells, rinsed with sterile water, and emptied into 1 ml of chemotaxis buffer. Serial dilutions were plated onto M9 minimal medium plates supplemented with 20 mM glucose and incubated overnight at 30 or 37°C. CFU counts were determined and corrected with the buffer control.

### RT-qPCR gene expression analysis.

Total RNA was extracted using a High Pure RNA isolation kit (Roche Diagnostics) and treated with Turbo DNase (Invitrogen). RNA quality was verified by agarose gel electrophoresis and quantified spectrophotometrically. Subsequently, cDNA was synthesized from 500 ng RNA using SuperScript II reverse transcriptase (Invitrogen) and 200 ng of random hexamer primers (Roche) following the instructions of the manufacturers. Quantitative PCR was performed using iQ SYBR green supermix (Bio-Rad) in a MyiQ2 thermal cycler (Bio-Rad). The following protocol was used: 95°C (5 min), 35 cycles of 95°C (10 s) and 61°C (30 s), and melting curve analysis from 55 to 95°C, with an increment of 0.5°C/10 s. Gene expression data were normalized to expression of the *rpoD* reference gene. The primers used are listed in Table S2.

### McpN-LBD crystallization and structure resolution.

Crystallization conditions were screened using the capillary counter-diffusion technique and commercially available crystallization kits GCB-CSK, PEG448-49, and AS-49 (Triana Science & Technology, Granada, Spain). The protein, maintained at 1.5 mg/ml in 20 mM Tris-HCl–200 mM NaCl (pH 7.5), was incubated at 4°C with 1.7 mM NaNO_3_, and the excess of NaNO_3_ was removed by centrifugation using Amicon concentrators (3-kDa cutoff). The protein-ligand complex was loaded into 0.2-mm-inner-diameter capillaries, and crystals of sufficient size appeared in 0.82 M K/phosphate–0.82 M Na/phosphate (0.1 M Na/HEPES, pH 7.5). Crystals were extracted from the capillary, flash-cooled in liquid nitrogen, and stored until data collection. Crystals were diffracted at beam line ID23-1 of the European Synchrotron Radiation Facility (ESRF). Data were indexed and integrated with XDS (76) and scaled with SCALA (77). Attempts at molecular replacement using homology models generated using the NarX sensor domain (PDB identifier [ID] 3EZI) and the NarQ sensor domain (PDB ID 5IJI) were unsuccessful. Phases were obtained using Arcimboldo (78) and searching for two helices that were 30 amino acids in length. Refinement was initiated with Refmac (79) and finalized with phenix.refine (80), tracking the quality with MolProbity (81). Refinement statistics and quality indicators are summarized in Table S3.

10.1128/mBio.02334-18.10 TABLE S3 Data collection and refinement statistics of the 3D structure of the McpN-LBD-nitrate complex. Values in parentheses represent the highest-resolution shell. Download Table S3, DOCX file, 0.02 MB. Copyright © 2019 Martín-Mora et al. 2019 Martín-Mora et al. This content is distributed under the terms of the Creative Commons Attribution 4.0 International license.

### Site-directed mutagenesis.

An overlapping PCR mutagenesis approach was used to construct the alanine substitution mutant McpN-LBD R61A. First, a NdeI/XhoI DNA fragment of pMcpN-LBD was cloned into the same sites of pCR2.1-TOPO and transformed into E. coli DH5α (*dam* positive [*dam*^+^]). Next, the resulting pCR-McpN-LBD plasmid was fully amplified by PCR using a complementary primer pair carrying the mutation. The parental plasmid was cleaved using DpnI, and plasmids with the desired mutation were recirculated with T4 DNA ligase (Roche). The presence of the mutation in the resulting plasmid, pCR-McpN-R61A, was confirmed by sequencing prior to cloning into the NdeI/XhoI sites of pET28(+) to generate pMcpN-R61A (Table 2).

### Data availability.

Coordinates and structure factors of McpN-LBD were deposited at the PDB with accession code 6GCV.
