# Supplementary figures and images for: The Molecular Mechanism of Nitrate Chemotaxis via Direct Ligand Binding to the PilJ Domain of McpN
Source: mBio. 2019 Feb 19;10(1):e02334-18. doi: 10.1128/mBio.02334-18 (PMC6381276; doi:10.1128/mBio.02334-18)

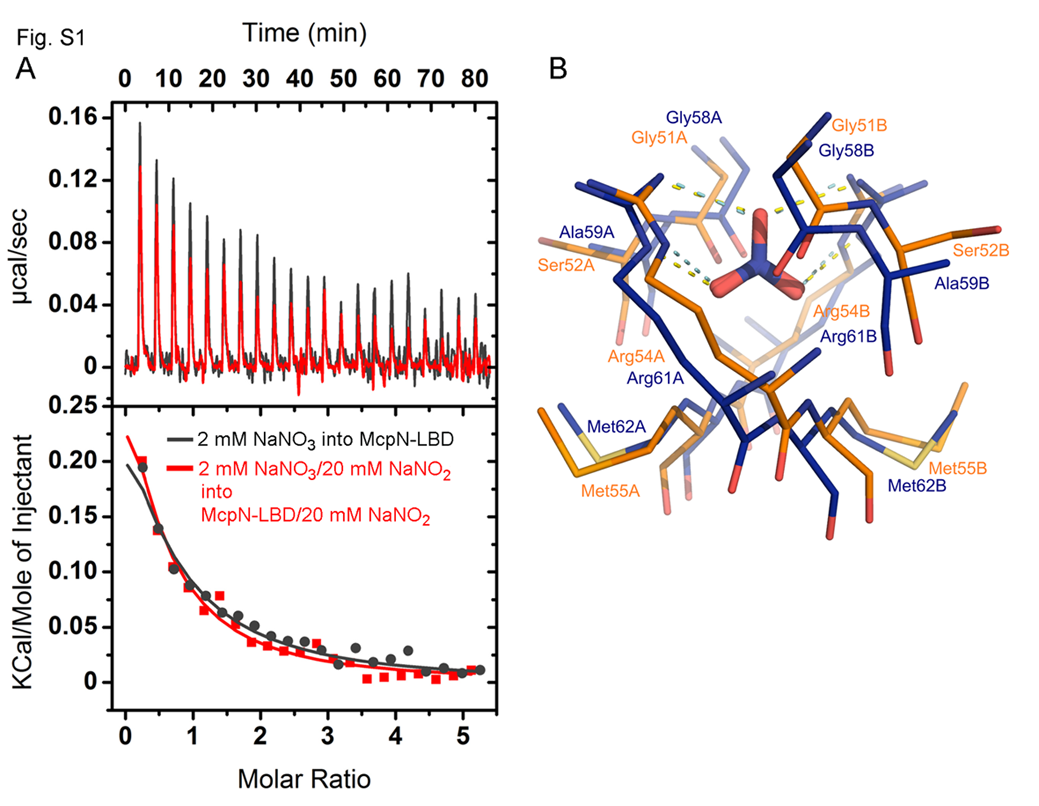

Supplement: FIG S1 [file mBio.02334-18-sf001.tif]

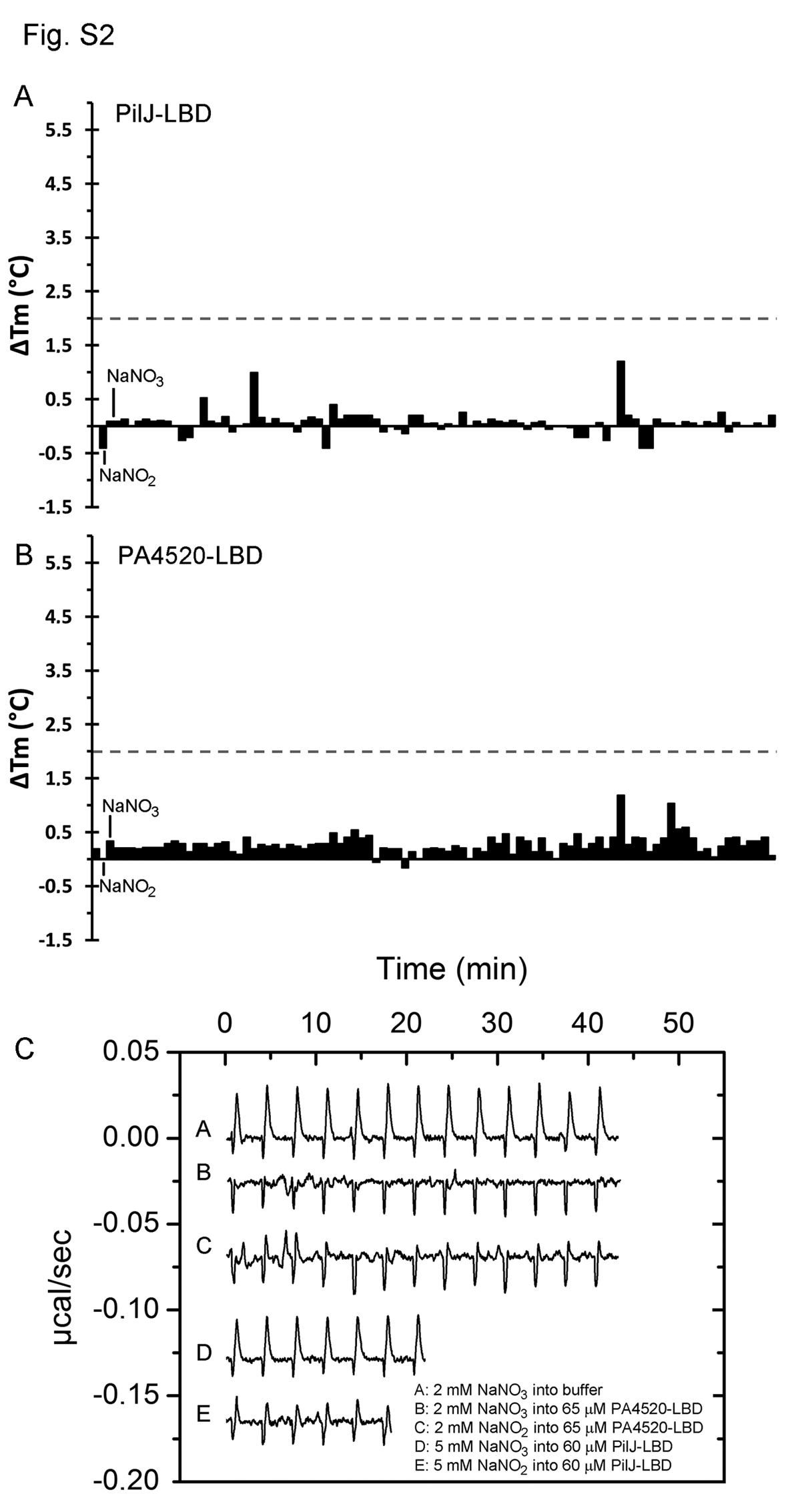

Supplement: FIG S2 [file mBio.02334-18-sf002.tif]

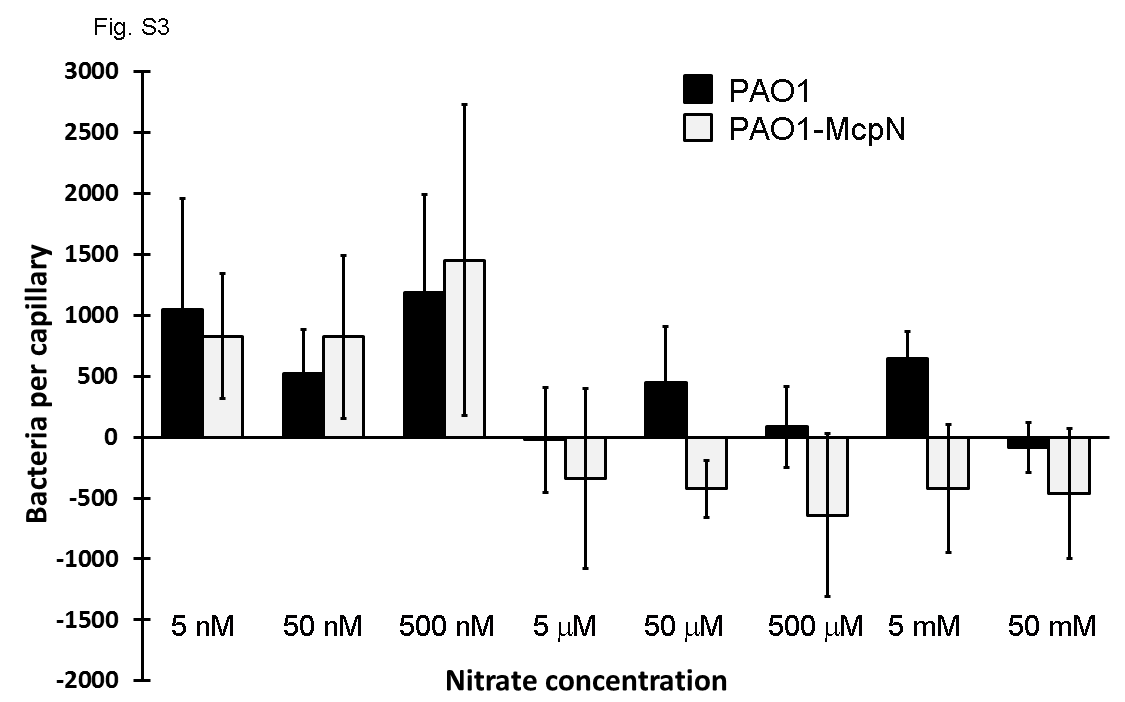

Supplement: FIG S3 [file mBio.02334-18-sf003.tif]

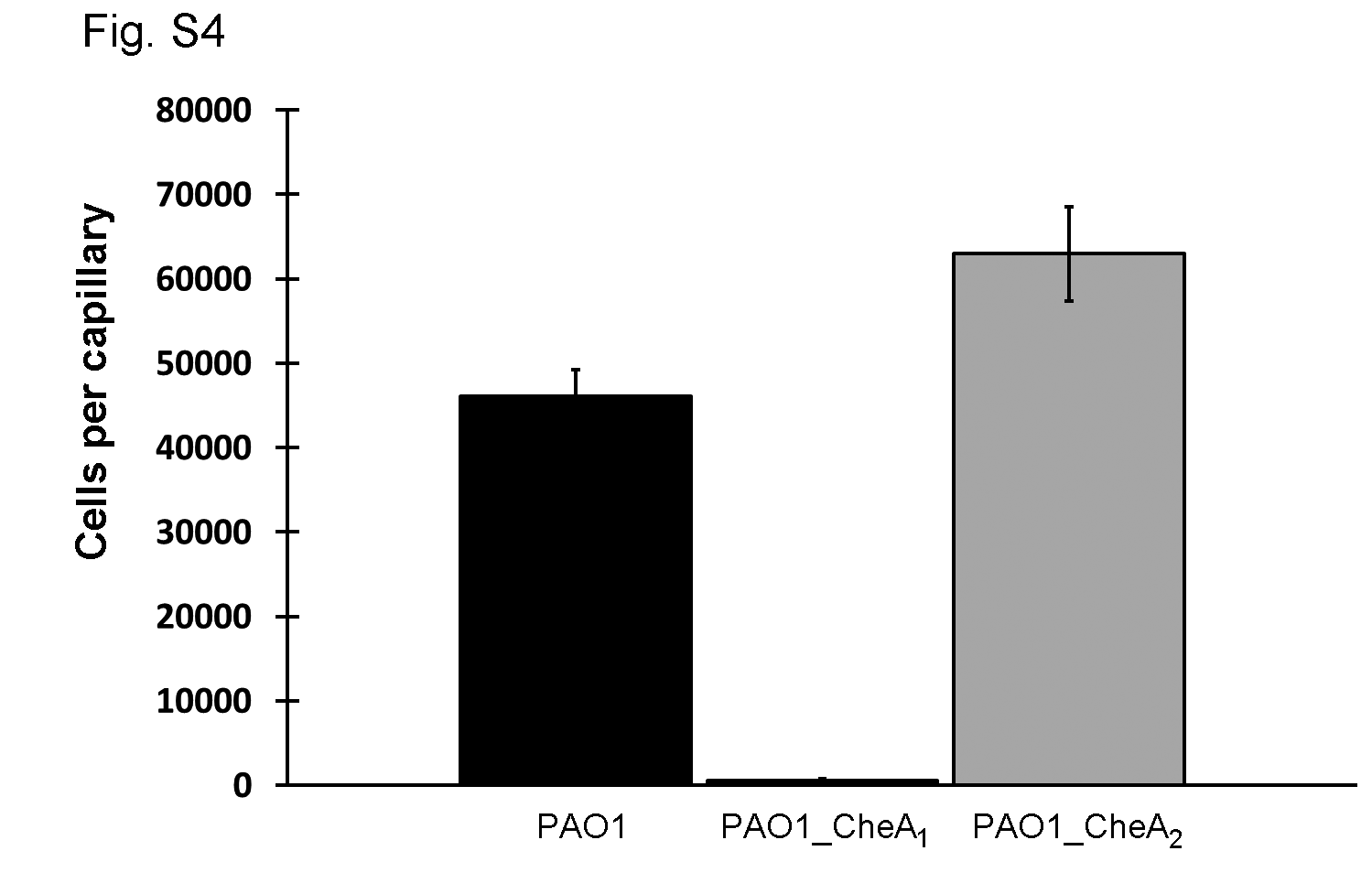

Supplement: FIG S4 [file mBio.02334-18-sf004.tif]

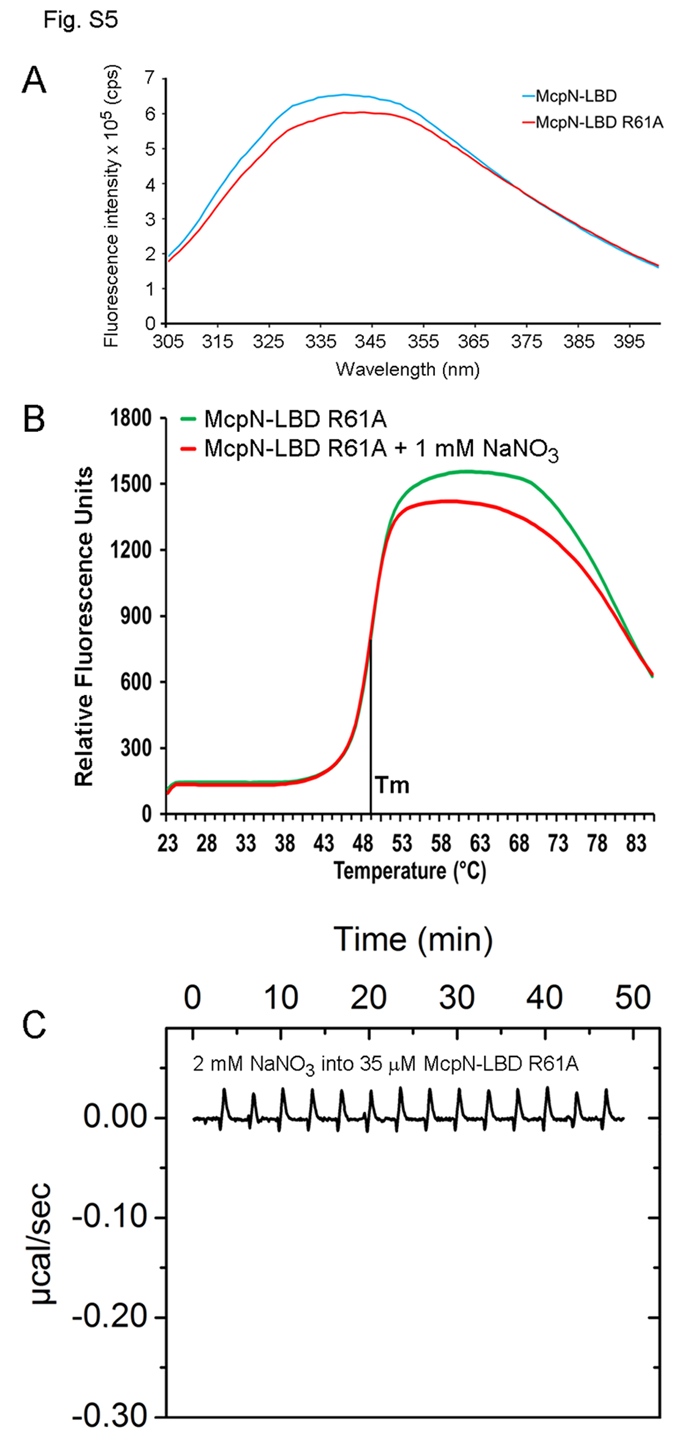

Supplement: FIG S5 [file mBio.02334-18-sf005.tif]

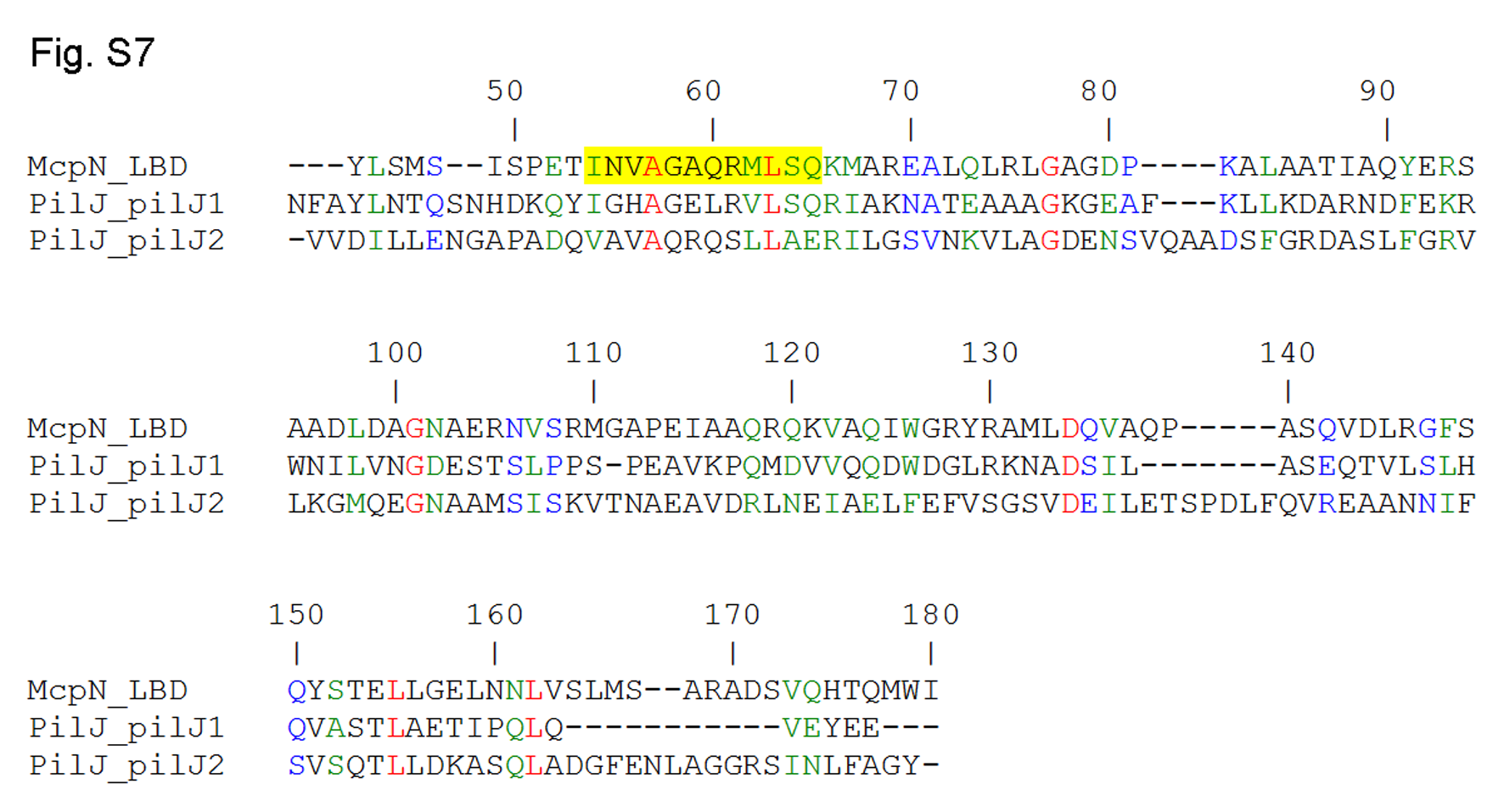

Supplement: FIG S7 [file mBio.02334-18-sf007.tif]
