# Supplementary material for: The Molecular Mechanism of Nitrate Chemotaxis via Direct Ligand Binding to the PilJ Domain of McpN
Source: mBio. 2019 Feb 19;10(1):e02334-18. doi: 10.1128/mBio.02334-18 (PMC6381276; doi:10.1128/mBio.02334-18)
Supplement: FIG S6 [file mBio.02334-18-sf006.pdf]

Fig. S6

	120 | 130 | 140 | 150 | 160 |
WP_083651730 [Photobacterium proteolyticum]	GIIYYGMEA-DAATINVAGRQRMLSQRVAKEVLLVQSGMEQ----QEGVNKTIKL
OLQ80001 [Photobacterium proteolyticum]	GIIYYGMEA-DAATINVAGRQRMLSQRVAKEVLLVQSGMEQ----QEGVNKTIKL
EDQ01086 [Shewanella benthica KT99]	AIIYNDMQA-DAATINIAGRQRMLSQLVAKEVLLLQFGVGR----DTGVKKAIEL
WP_040571760 [Shewanella benthica]	AIIYNDMQA-DAATINIAGRQRMLSQLVAKEVLLLQFGVGR----DTGVKKAIEL
WP_022940319 [Psychromonas hadalis]	SIIYSSMEA-DATTINVAGKQRMLSQRIAKEVLLVQFSSLE----KQQVQQTITQ
WP_104417027 [Marinobacter persicus]	SFLYFSTDPGEARSLNVAGAQRMLSQRVAKEVQMVAAGVEE----PAQAQKTIEQ
KXS52579 [Marinobacter sp. T13-3]	SFLYFSTDPGEARSLNVAGAQRMLSQRVAMEVQMVAAGVED----PAQAQKTIEQ
KXS51160 [Marinobacter sp. T13-3]	SFLYFSTDPGEARSLNVAGAQRMLSQRVAKEVQMVAAGVEE----PAQAQKTIEQ
WP_104425587 [Marinobacter persicus]	SFLYFSTDPGEAKSLNVAGAQRMLSQRVAKEVQMVAAGVEE----SSQAQQTIDQ
WP_104416353 [Marinobacter persicus]	SFLYFSTDPGEAKSLNVAGAQRMLSQRVAKEVQMVAAGVEE----SSQAQQTIDQ
PKM00178 [Gammaproteobacteria bacterium HGW]	-----SSK--DASQIDMAGAQRMLSQKMMKEALLAAQGIGS----PADVDRTIQR
PKM12203 [Gammaproteobacteria bacterium HGW]	AVLFMSAK--DASQIDMAGAQRMLSQKMMKEALLAAQGVGD----KAAVDKTIQR
CJK46151 [Streptococcus pneumoniae]	GVLFMSSK--DASQIDMAGAQRMLSQKMMKEALLAAQGIGS----PADVDKTIQR
McpN [P. aeruginosa PAO1]	VALYLSMSI-SPETINVAGAQRMLSQKMAREALQLRLGAGD----PKALAATIAQ
OUC50533 [Eggerthia catenaformis]	VALYLSMSI-SPETINVAGAQRMLSQKMAREALQLRLGAGD----PKALAATIAQ
SAJ26551 [Enterobacter cloacae]	VALYLSMSI-SPETINVAGAQRMLSQKMAREALQLRLGAGD----PKALAATIAQ
PKM29122 [Gammaproteobacteria-12]	VALYLSMSV-SPETINIAGAQRMLSQKMTKEALLQREGVLP----AATLEATMAQ
WP_027848104 [Marinospirillum minutulum]	VSLYMSLSV-SPETINVAGAQRMLSQKMTKEALLITQGVEE----RKVLDSTINS
WP_027850591 [Marinospirillum insulare]	ISLYMSLSV-SPETINVAGAQRMLSQKMTKEALLIIGKVEN----KTVLDKTVAS
WP_072325584 [Marinospirillum alkaliphilum]	VSLYMSLSV-SPETINVAGAQRMLSQKMTKEALLITQGASD----RGTLDATMRQ
WP_091963700 [Marinospirillum celere]	ASLFMSLSV-SPETINVAGAQRMLSQKMTKEALLLTQGVGD----QATLNATQRQ
WP_068999334 [Terasakiispira papahanaumokuakeensis]	VSLFLSMSV-SPQTINVAGAQRMLSQKMTKEVLLLEVGAVD----QQTLQTTMQR
SFX75036 [Marinospirillum alkaliphilum DSM 21637]	ISLYLSMSV-SPETINVAGAQRMLSQKITRDVLLVVQGAEQ----QQVLNRTIQR
WP_084662276 [Marinospirillum alkaliphilum]	ISLYLSMSV-SPETINVAGAQRMLSQKITRDVLLVVQGAEQ----QQVLNRTIQR
WP_086481606 [Oceanospirillum sanctuarii]	VSLYLSMAI-DPQTINIAGRQRMLSQKMAKEALLVAAQVEN----KSTLQKTMQL
WP_078320434 [Oceanospirillales]	IFLYLSMAI-DPQTINIAGRQRMLSQKMAKEALLVASQVEN----KSTLQKTMQL
WP_102043389 [Oceanospirillum maris]	VSLYLSMAI-DPQTINIAGRQRMLSQKMAKEALLVAAQVEN----KTTLQKTMQL
WP_028300494 [Oceanospirillum beijerinckii]	VSLYLSMAI-NPQTINIAGRQRMLSQKIAKEALLVAAQVEN----QNTLQKTMQL
WP_078743715 [Oceanospirillum multiglobuliferum]	VALYMSMAI-NPQTINVAGRQRMLSQKIAKEALLVAAKIEN----EAVLRKTMQL
EAR59890 [Oceanospirillum sp. MED92]	ISLYFSMAI-NPQTINMAGRQRMLSQKIAKEALLVAAQVEQ----QSTLKKTMEL
WP_083774892 [Neptuniibacter caesariensis]	ISLYFSMAI-NPQTINMAGRQRMLSQKIAKEALLVAAQVEQ----QSTLKKTMEL
WP_093308400 [Pseudospirillum japonicum]	IALYLSMSI-NPQTINIAGRQRMLSQKMAKEALLVAVNIEN----TSNLQATMQM
WP_051252630 [Ferrimonas kyonanensis]	VMLYATSGN-SAEMINVAGAQRMLSQRYAKEALLVVQGGVD----HAALDKTVAR

WP_075186461 [Alteromonadales bacterium BS08]	LSLYISMSV-SANTVDIAGRQRMLSQRLAKEAFLVVNGAEQ----MEAVRSTIGL
WP_045855660 [Alteromonadaceae bacterium Bs12]	GSMYLTLDI-TADTVDVAGRQRMLTQRLAKEAFLVANEAEP----QSVVQETITE
WP_018274184 [Teredinibacter turnerae]	FSLYMTMQA-SADTVNIAGRQRMLSQRLAKEALLVGQQAMD----KSALEQTIEL
WP_019606173 [Teredinibacter turnerae]	FSLYMTMQA-SADTVNIAGRQRMLSQRLAKEALLVGQQAMD----KSALEQTIEL
WP_045827023 [Teredinibacter sp. 991H.S.0a.06]	FSLYMTMQA-SADTVNIAGRQRMLSQRLAKEALLVAQQAMD----KSALEQTIEL
WP_028885168 [Teredinibacter turnerae]	FSLYMTMQA-SADTVNIAGRQRMLSQRLAKEALLVGQQAMD----KSALEQTIEL
WP_028881853 [Teredinibacter turnerae]	FSLYMTMQA-SADTVNIAGRQRMLSQRLAKEALLVAQQAMD----KSALEQTIEL
WP_018415357 [Teredinibacter turnerae]	FSLYMTMQA-SADTVNIAGRQRMLSQRLAKEALLVAQQAMD----KSALEQTIEL
WP_015819270 [Teredinibacter turnerae]	FSLYMTMQA-SADTVNIAGRQRMLSQRLAKEALLVTQQAMD----KSALEQTIEL
WP_028876398 [Teredinibacter turnerae]	FSLYMTMQA-SADTVNIAGRQRMLSQRLAKEALLVTQQAMD----KSALEQTIEL
WP_019602011 [Teredinibacter turnerae]	FSLYMTMQA-SADTVNIAGRQRMLSQRLAKEALLVTQQAMD----KSALEQTIEL
WP_011467154 [Saccharophagus degradans]	ISLYLSMSS-SANTVDVAGRQRMLSQRLAKEALLVGAGVES----RATMQSTIDL
WP_082067039 [Teredinibacter sp. 1162T.S.0a.05]	GSLYLTMSA-SAETVNIAGRQRMLSQRLAKEALLIRTGAEN----EAIAQQTIAL
WP_027328238 [Marinimicrobium agarilyticum]	GVSYLSLSA-TAETVDVAGRQRMLIQRLAKEAMLTEQGALD----ASGLNATIEL
WP_076717438 [Motiliproteus sp.MSK22-1]	GSLYLTMSA-SAETINVAGRQRMLSQRLAKETLMVVQGVEQ----KVAVEKTMQL
WP_076714176 [Motiliproteus sp.MSK22-1]	VSLYFSLSN-DAATINEAGRQRMLSQRLAKEAMMVVQGVEE----KAVLEKTIKL
WP_081475260 [Marinobacterium stanieri]	VSVWMTGSD-DATAINVAGKQRMLSQRLAKETLLIQQGAVD----TGLAQATIRS
WP_076462706 [Marinobacterium stanieri]	VSVWMTGSD-DATAINVAGKQRMLSQRLAKETLLIQQGAVD----TGLAQATIRS
WP_091825072 [Marinobacterium georgiense]	AAVYLSDNT-DASAINVAGAQRMLSQKMAKEVLLLSQDVGD----RSGVTKTMNA
PKM43282 [Gammaproteobacteria bacterium HGW]	ASLFYGFGT-DATSINVAGAQRMLSQKVAKEALLAGQGGES----RETVYATLAQ
PKM45884 [Gammaproteobacteria bacterium HGW]	ASLYFSIST-DATSINVAGAQRMLSQKVAKEALLAGQGVES----RDTVLATIRQ
OOZ41054 [Solemya velesiana gill symbiont]	ASLYFSLGS-DATSINVAGRQRMLSQKVAKEAMLAAQKVES----LQAVEKTIAL
WP_078483146 [Solemya velesiana gill symbiont]	ASLYFSLGS-DATSINVAGRQRMLSQKVAKEAMLAAQKVES----LQAVEKTIAL
WP_083220704 [Candidatus Thiodiazotropha endolucinida]	VSLFTSLGS-DATAINVAGRQRMLSQKVAKEALLAAQSIES----RDTVNNTIAL
ODJ87354 [Candidatus Thiodiazotropha endolucinida]	VSLFTSLGS-DATAINVAGRQRMLSQKVAKEALLAAQSIES----RDTVNNTIAL
WP_084594071 [Arhodomonas aquaeolei]	ASLYFSLGS-SATAVDIAGRQRMLSQRVAKEAVMAVQGVGS----REDVEKTIAL
WP_029132713 [Sedimenticola selenatireducens]	ITIYLSLGV-NSNSIDVAGRQRMLSQRLAKEAMLVAQQAES----REVMEKTINL
PLX63594 [Sedimenticola selenatireducens]	ITIYLSLGV-NSNSIDVAGRQRMLSQRLAKEAMLVAQQAES----REVMEKTINL
WP_057957096 [endosymbiont of Ridgeia piscesae]	ASIYWSLDS-DAAAINIAGRQRMLSQRVAKEALMVVQGVES----PAVVQKTLAL
WP_067616512 [Dissulfuribacter thermophilus]	LKTVYGQRG-DAVAINIAGRQRMLTQKMTKEALFFNSTQNDK--WKSSLDATIVL
WP_083779049 [Desulfarculus baarsii]	IVLLGLQET-DGQVVNIAGRQRMLSQRMTKEALILASGDRAAAKN---LAATLEL
ADK86400 [Desulfarculus baarsii DSM 2075]	IVLLGLQET-DGQVVNIAGRQRMLSQRMTKEALILASGDRAAAKN---LAATLEL
WP_053111464 [Desulfocarbo indianensis]	IILLGMQEA-DGVVVDIAGRQRMLSQKMTKEALLLLSSGGNTEKDRKELKATADL
WP_072909408 [Malonomonas rubra]	VFVLNGQKD-DSTVVNIAGRQRMLSQKMSKEALSIAAGLEVSSNR-DSLKQTADL
ENN95934 [Methanocaldococcus villosus KIN24-T80]	TILLKDMEH-DANIINIAGKQRMLIQKMSKEAFMIALGNL---EMKKELIKTAQE
WP_017981124 [Methanocaldococcus villosus]	TILLKDMEH-DANIINIAGKQRMLIQKMSKEAFMIALGNL---EMKKELIKTAQE
WP_079710389 [Paraliobacillus ryukyuensis]	NMFLTEQET-DATIINVAGKQRMLIQKMSKEALIVSDGN----GSADDLVETINL

```
ODS31255 [Candidatus Scalindua rubra]                ---MLEKKASDPVKINLAGKQRMLTQKMSKEAIALSQG----IGSTESLEKTANL
ODS31259 [Candidatus Scalindua rubra]                TISFLNKQKADGVVINLAGKQRMLTQKMSKEALAVSQG----TGSKESLEKTADL
ODS31256 [Candidatus Scalindua rubra]                VIVLMNSQKDDGAVINLAGKQRMLTQKMSKEAIALSQG----IGSKQSLVKTINL
DAB34330 [Sulfurospirillum sp. UBA12182]            NVIMNDKSKKDSLIINIAGKQRMLTQKMSKEIFYLKQKD---SIDFRELNSAVDE
DAB32648 [Sulfurospirillum sp. UBA11407]            NVIMNDKSKKDSLIINIAGKQRMLTQKMSKEIFYLKQKD---SIDFRELNSAVDE
OQY52690 [Beggiatoa sp. 4572_84]                    ACGGGVTKQEMGVIMDLAGKQRMFTQKMTKEILLIAKGINV-AENKKKLRQTAIL
OAD20060 [Candidatus Thiomargarita nelsonii]        ------------MSIDLAGKQRMLTQKMSKEILLIAKNIDR-DDNKKNLCETAAL
KHD07211 [Candidatus Thiomargarita nelsonii]        AY--AATQQEMATTINLAGKQRMLTQKMSKEILLIAKGINV-AANKKNLQKTAAL
WP_083760414 [Sulfurovum sp. NBC37-1]              AV--AQTKQQSGVVINLAGKQRMLTQKMSKEALYIAKGIDA-EANTENLKKTAAL
BAF71977 [Sulfurovum sp. NBC37-1]                  AV--AQTKQQSGVVINLAGKQRMLTQKMSKEALYIAKGIDA-EANTENLKKTAAL
WP_103922382 [Thiotrichales bacterium HS_08]       AQ--ASSKTEMGKVINLAGKQRMLTQKMSKEALFIAKGVDA-TGNQGNLKKTANL
WP_038034074 [Thermopetrobacter sp. TC1]           NAIVEQSSAELAVQINLSGRQRMLTQKMSKEAFLVALGVDP-EENRQNAARTAAL
OQX74572 [Campylobacteraceae bacterium 4484_4]     SF--ALTTKQLAVSINLAGKQRMLTQKMTKEALLIKAGVEK-EQNLKKLEATSTL
OGX22015 [Omnitrophica WOR_2 bacterium GWF2_43_52] GFC-APTPEQWGIIMNISGRQRMLSQKMSKEALLAIAGINP-DENLKKLAESMKL
OGR47178 [Elusimicrobia bacterium GWA2_66_18]      SS--AASEKEFAKVINVAGRQRMLSQKMAAEFLLMKLGIAA-EDNKKKMDADIST
OGR71769 [Elusimicrobia bacterium GWC2_65_9]       SS--AASEKEFAKVINVAGRQRMLSQKMAAEFLLMKLGIAA-EDNKKKMDADIST
PIQ95337 [Nitrospinae bacterium CG11]              TVFTLENQKLDGNVINLAGKQRMLTQKLSKSIMELQLG--D-LSKTGEIGQIKTE
WP_037929253 [Sulfitobacter pseudonitzschiae]      TAQVAASGSAVVVRVDISGRQRMLSQRMAMASCFVMGDVETEKN-IKNAHQAYDL
```

```
                      175       185       195       205       215
                        |         |         |         |         |
WP_083651730   FESSMNLLRNGDKEQGISAPKTPEIEAQLSKVNELWLEYRAGIQKLLQIDESQLR
OLQ80001       FESSMNLLRNGDKEQGISAPKTPEIEAQLSKVNELWLEYRAGIQKLLQIDESQLR
EDQ01086       FESSMNMLLNGDTKRGISAPMTPEIKSRLGKVNKLWSKYREGIQALLLLGDKDQH
WP_040571760   FESSMNMLLNGDTKRGISAPMTPEIKSRLGKVNKLWSKYREGIQALLLLGDKDQH
WP_022940319   FESSMNKLINGDESAGIHAFSDVAIKTQLLKVKDTWQEYRINIEQILQLSPFDLA
WP_104417027   WERAHGWLLNGSEEAGVPAVKDPEIRAQLESVFALWQEYRPALVDYMNAPDT---
KXS52579       WERAHGWLLNGSEEAGVPAVKDPDIRAQLESVFALWQKYRPALVDYMNAPDT---
KXS51160       WERAHGWLLNGSEEAGVPAVKDPEIRAQLEHVLTLWQKYRPALVDYMNAPDT---
WP_104425587   WERAHGWLLNGSEEAGVPAVTDTAIRAQLEHVLTLWQDYRPALVEYMEAPDT---
WP_104416353   WERAHGWLLNGSEEAGVPAVTDTAIRAQLEHVLTLWQDYRPALVEYMEAPDT---
PKM00178       FEQSHRLLLNGDRAQGIAPVELASAKPHLQRVDQIWQRYRPA-VQALAAGQS---
PKM12203       FEQSHRLLLNGDRAQGIAPVELTSAQPHLQRVDQIWQRYRPA-VQALAAGQS---
CJK46151       FEQSHRLLLNGDSAQGIVRVELASAQPYLQRVDQIWQRYRPA-VQALAAGQS---
McpN_LBD       YERSAADLDAGNAERNVSRMGAPEIAAQRQKVAQIWGRYRAMLDQVAQPAS----
OUC50533       YERSAADLDAGNAERNVSRMGAPEIAAQRQKVAQIWGRYRAMLDQVAQPAS----
```

```
SAJ26551       YERSAADLDAGNAERNVSRMGAPEIAAQRQKVAQIWGRYRAMLDQVAQPAS----
PKM29122       FDAAHRDLLSGNAVRNISAIAEPGIQAQMNKVGGLWQGFRTQLQRVVA-GD----
WP_027848104   FERAHQDLLVGNRERNITAFDDEEIHQQMQQVDQLWQQMKARLEQAVNKQD----
WP_027850591   FETAHKDLIAGNRDRDITAFDDSEIQQQMQQVDQLWQQMKVRLDQALVQQD----
WP_072325584   FEQAHRDLLQGNRERNITAFDDPAIRQQMQRVDQLWQQMKTRLDATLTNPG----
WP_091963700   FEQAHQDLIQGNPERNISAIDDPEIQQQMQRVDDLWQQIQDSLQEVVNNPN----
WP_068999334   FETAHRDLMAGNPAKNIAVFDDPVIQRQMQTVDQLWQQMASLINRQLQDPQ----
SFX75036       FDAAHRDLVQGNPERSISRINDPVVQQQLQQVEVSWQEFQGVIQNYLKQPL----
WP_084662276   FDAAHRDLVQGNPERSISRINDPVVQQQLQQVEVSWQEFQGVIQNYLKQPL----
WP_086481606   FESSHKDIINGNKAQGMNPITDKEILGQMQKVESLWKDYSKTLLQYADNPT----
WP_078320434   FERSHKDIINGNKAQGMNPTTDPSILSQMQIVENLWSGYSKILLQYTDSPS----
WP_102043389   FEQSHDHILNGNKSLSMNPIEDPEIVAQMQRVQDLWRSYSITLLEYAERPD----
WP_028300494   FERSHKDIIHGNTELGMNPINDKAILAQMQKVEGLWQSYKGMINRYASTPT----
WP_078743715   FEQSHRDIVNGNVEQGMNPITNPAVLSQMQKVEGLWRDYSALVERYIANPN----
EAR59890       FESSHKKIMLGNEELGMNAIKDSEILKQMQHVETLWATYKGVIESHITQPS----
WP_083774892   FESSHKKIMLGNEELGMNAIKDSEILKQMQHVETLWATYKGVIESHITQPS----
WP_093308400   FDRSHQDIIQGNTQQGMNPITDEDILQQMAVVGRLWQDYRELLLAYVQAPS----
WP_051252630   FERAHRLLLEGDANRGLPPVSEPAIVQQLNRVGEFWSSYRQQVDAYLMAPS----
WP_075186461   FENSHRDLLAGNRNADIQPPATKAIESQLKKVEGVWNGYQQSVTRYINSQQ----
WP_045855660   FERSHRDLMMGNKRAGIAIP-TSEVLAQLKEVERVWRQYKLTVAAYIRSKN----
WP_018274184   FEQSHNRLLYGSPENGILAPQTVKIFDKLKVVGGMWENYKIAIRTYATTQD----
WP_019606173   FEQSHNRLLYGSPENGILAPQTVKIFDKLKVVGGMWENYKIAIRTYASTQD----
WP_045827023   FEQSHNRLLYGSPENGILAPQTVKIFDKLKVVGGMWENYKIAIRTYAATQD----
WP_028885168   FEQSHNRLLYGSPENGILAPQTVKIFDKLKVVGGMWENYKIAIRTYATTQD----
WP_028881853   FEQSHNRLLYGSPENGILAPQTVKISDKLKAVGGMWENYKIAIRTYVATKD----
WP_018415357   FEQSHNRLLYGSPENGILAPQTVKISDKLKAVGGMWENYKIAIRTYVASKD----
WP_015819270   FEQSHNRLLYGSPENGILAPQTVKIFDQLKVVDGLWENYKIAIRTYVASKD----
WP_028876398   FEQSHNRLLYGSPENGILAPQTVKIFDQLKVVDGLWENYKIAIRTYVASKD----
WP_019602011   FEQSHNRLLYGSPENGILAPQTVKIFDQLKVVDGLWENYKIAIRTYVASKD----
WP_011467154   FERSHRKLISGDQSDDIHPPATQEIKQALVTVEKQWAEYKRLVNHYVSAKD----
WP_082067039   FESSHQALMNGDKDKGIHAPESAEIKQQLQKVEALWLEYKKGIASLIAGED----
WP_027328238   FEQSHQNLLNGNESAGIQPPQTAEINQSLLNVLQEWNQYKAIIFDYIGADP----
WP_076717438   FESSHQDLINGNPSTGIVAPATPQISQQLEHVGSLWTDYKQTINNYLVDAS----
WP_076714176   FSDSHLHLINGNPESGISAPANAEIRKQLEHVGQLWQHYQSAISNYLENPN----
WP_081475260   FEAAHKALLNGNADMGIKAASEVDIRKQLEHVGTLWRDYRATVEQLVSGTDGN--
WP_076462706   FETAHRALLNGNAGLGIKAASEVDIRKQLEHVGTLWRDYRATVEQLVSGSDGN--
WP_091825072   FESAHQALLNGSAEQGIKAAEDAEIRSRLEQVGTLWNSYRATLNQYLQQPD----
```

```
PKM43282       FESVHQALLQGDAQRDIAVVTDTAVRAQLQKVGQVWKEYRQEIVAYVEQPA----
PKM45884       FEGAHRALLEGDAQRGMRAVKDAAVRTQLQKVEQLWQAYKQDILAYIEQPD----
OOZ41054       FESSHNALLQGDKKQKIDAVKAKPIIDQLKHVEQLWGKYKVHILSYSNNPT----
WP_078483146   FESSHNALLQGDKKQKIDAVKAKPIIDQLKHVEQLWGKYKVHILSYSNNPT----
WP_083220704   FESSHQNLLNGDQNAGIQAVDDPVVRGQLNKVEGLWITYRESIDAYLENPS----
ODJ87354       FESSHQNLLNGDQNAGIQAVDDPVVRGQLNKVEGLWITYRESIDAYLENPS----
WP_084594071   FESSHRKLLQGDPGMGIEPVQDPAIRDQLREVQSLWQRYRGDIQAYLDEPT----
WP_029132713   FESSHKALFEGDEAQNILPVKDALILEQLKLVEQLWSGYKQVITQYAASPD----
PLX63594       FESSHKALFEGDEAQNILPVKDALILEQLKLVEQLWSGYKQVITQYAASPD----
WP_057957096   FEDSQHRLLLGDPEAGITPPMNEAIRQQLAKTSQLWSAYRRHLEAYLATPS----
WP_067616512   FEKSLNGLAVGDAELGLSGTTDKGVQEELNKLQQMWIPFKKALYIITDTSSSK--
WP_083779049   FDASLRGLIEGDAAMGLPPTDDSRIAAQMQKVAALWGPFRQSAEIVLRAGGSADP
ADK86400       FDASLRGLIEGDAAMGLPPTDDSRIAAQMQKVAALWGPFRQSAEIVLRAGGSADP
WP_053111464   FDRSLQGLIGGDAAMGLPPTTNPDILAQMKTVSELWKPFHANLTGVTITTDINSP
WP_072909408   FASSLNSLINGDEKLNLPPTQNPQILSQMRQVEVLWDNFSPHIDTFVNP-ASTEA
ENN95934       FDKNLNDLINGNEERGITPAPP-IVKAQLLKVKSMWSEFYKNILIIYEK-DPSDP
WP_017981124   FDKNLNDLINGNEERGITPAPP-IVKAQLLKVKSMWSEFYKNILIIYEK-DPSDP
WP_079710389   FDEAIEGLMGGSKSLGLPKMPSEEVANQLQSVELLWQPFKENLQMIAN--DNGN-
ODS31255       FDKTLRGLISGDEELRLSPTKDPKIISQLNHIQGLWQDFRANLNAVLANPTGA--
ODS31259       FDKTLKGLISGDKELGLPATKNIEILSQLNQVQKLWKDLHANLDVVLANSDVT--
ODS31256       FDKTLKGLVSGDSELNLPATSNPEILGQLNHVQKLWKDLHANLSIVLANSDVT--
DAB34330       FSENLKDLLEGNSVKGIYHPQDEKIEAKLQKVQKIWFPFKEKIEALKKLIQEN--
DAB32648       FSENLKDLLEGNSVKGIYHPQDEKIEAKLQKVQKIWFPFKEKIEALKKLIQEN--
OQY52690       FNKTLIGLFDGDSELGLVKVENPHIVQQLNKVADLWREFRNNVDAVLRG-NTS--
OAD20060       FNQTLIGLIEGDSELGLVETEKPAIKQQLYKVVELWNKFRINVDAVLMG-NTS--
KHD07211       FERTLKGLLNGDARLGLVKTENAAIVKQLKKVGRLWGKFRQNVKAVLAG-NTS--
WP_083760414   FDKTLKGLIGGDSSLNLPKTDNKEILAQLQKVTDLWVPFKANIDKVIAG-KAD--
BAF71977       FDKTLKGLIGGDSSLNLPKTDNKEILAQLQKVTDLWVPFKANIDKVIAG-KAD--
WP_103922382   FDRTLKGLKDGDADLGLPKTTDAGILAQLDVVAKLWITFKGNIDAVLAG-KTS--
WP_038034074   FERTLKGLMQGDEALKLAPAPNEKILAQLKKVEGLWRRFKPLIEKVAAG-DVS--
OQX74572       FDRTLKGLIKGDEGLKLKPCQNAEVQKQLGVVQQLWKPFRQNIMRVIQG-KAD--
OGX22015       FETSHHALTEGDAAMNIPACEFPDISEQLEKVSILYAELDKIFDKMVEGEKAD--
OGR47178       FDKSLASLSNGDGEAGIPAPPNEQISRQFAQVKLLWGSYVRALQSAGTS------
OGR71769       FDKSLASLSNGDGEAGIPAPPNEQISRQFAQVKLLWGSYVRALQSAGTS------
PIQ95337       FEGVLSGLKRGDTEKGLVAAETPEIMAMLEATEKLWLPFAERVDKVASLWPGI--
WP_037929253   FSQTQGVLRHGGTRDNLEPERDPQVLALLDQSDEIFDTYGRAVLQVTHQDLQ---
```
