# Supplementary material for: The Molecular Mechanism of Nitrate Chemotaxis via Direct Ligand Binding to the PilJ Domain of McpN
Source: mBio. 2019 Feb 19;10(1):e02334-18. doi: 10.1128/mBio.02334-18 (PMC6381276; doi:10.1128/mBio.02334-18)
Supplement: TABLE S1 [file mBio.02334-18-st001.docx]

**Table S1)**

| **Species** | **Taxonomic group (Phylum/Class/Order)** | **Source/lifestyle/comments** | **References/Source** |
| --- | --- | --- | --- |
| *Photobacterium proteolyticum* | Proteobacteria/Gammaproteobacteria/Vibrionales | Isolated from the ocean sediment of Laizhou Bay, PR China, optimal growth in 2-3 % (w/v) NaCl | (1) |
| *Shewanella benthica* | Proteobacteria/Gammaproteobacteria/Alteromonodales | Isolated from the abyssal South Pacific Ocean, piezophilic | (2) |
| *Psychromonas hadalis* | Proteobacteria/Gammaproteobacteria/Alteromonodales | Collected from the bottom of the Japan Trench, piezophilic | (3) |
| *Marinobacter persicus* | Proteobacteria/Gammaproteobacteria/Alteromonodales | Isolated from the hypersaline lake Aran-Bidgol in Iran, halophilic | (4) |
| *Streptococcus pneumoniae* | Firmicutes/Bacilli/Lactobacillales | Human pathogen, particularly upper airways infections | (5) |
| *Eggerthia catenaformis* | Firmicutes/[Erysipelotrichi](https://en.wikipedia.org/wiki/Erysipelotrichi)a/[Erysipelotrichales](https://www.ncbi.nlm.nih.gov/Taxonomy/Browser/wwwtax.cgi?mode=Undef&id=526525&lvl=3&lin=f&keep=1&srchmode=1&unlock) | Isolated from human dental abscess, causes bacteremia | (6) |
| *Enterobacter cloacae* | Proteobacteria/Gammaproteobacteria/Enterobacterales | Human pathogen, urinary tract infections, nosocomial infections | (7) |
| *Marinospirillum minutulum* | Proteobacteria/Gammaproteobacteria/[Oceanospirillales](https://en.wikipedia.org/wiki/Oceanospirillales) | Isolated from Japanese fermented brine, halophilic | (8) |
| *Marinospirillum insulare* | Proteobacteria/Gammaproteobacteria/[Oceanospirillales](https://en.wikipedia.org/wiki/Oceanospirillales) | Isolated from fermented fish brine in Japan, halophilic | (9) |
| *Marinospirillum alkaliphilum* | Proteobacteria/Gammaproteobacteria/[Oceanospirillales](https://en.wikipedia.org/wiki/Oceanospirillales) | Isolated from Haoji soda lake in China, halophilic, alkaliphilic | (10) |
| *Marinospirillum celere* | Proteobacteria/Gammaproteobacteria/[Oceanospirillale](https://en.wikipedia.org/wiki/Oceanospirillales)*[s](https://en.wikipedia.org/wiki/Oceanospirillales)* | Isolated from Mono Lake (USA), halophilic, alkaliphilic | (11) |
| *Ferrimonas kyonanensis* | Proteobacteria/Gammaproteobacteria/Alteromonadales | Isolated from the Tokyo Bay, | (12) |
| *Oceanospirillum sanctuarii* | Proteobacteria/Gammaproteobacteria/[Oceanospirillales](https://en.wikipedia.org/wiki/Oceanospirillales) | Sediment sample collected at the Coringa Wildlife Sanctuary, Indian Ocean | (13) |
| *Oceanospirillum maris* | Proteobacteria/Gammaproteobacteria/[Oceanospirillales](https://en.wikipedia.org/wiki/Oceanospirillales) | Marine bacterium | (14) |
| *Oceanospirillum beijerinckii* | Proteobacteria/Gammaproteobacteria/[Oceanospirillales](https://en.wikipedia.org/wiki/Oceanospirillales) | Marine bacterium | (14) |
| *Oceanospirillum multiglobuliferum* | Proteobacteria/Gammaproteobacteria/[Oceanospirillales](https://en.wikipedia.org/wiki/Oceanospirillales) | Marine bacterium | (14) |
| *Neptuniibacter caesariensis* | Proteobacteria/Gammaproteobacteria/[Oceanospirillales](https://en.wikipedia.org/wiki/Oceanospirillales) | Isolated from a surface water sample from the eastern Mediterranean Sea, slightly halophilic | (15) |
| *Pseudospirillum japonicum* | Proteobacteria/Gammaproteobacteria/[Oceanospirillales](https://en.wikipedia.org/wiki/Oceanospirillales) | Marine bacterium | (14) |
| *Terasakiispira papahanaumokuakeensis* | Proteobacteria/Gammaproteobacteria/[Oceanospirillales](https://en.wikipedia.org/wiki/Oceanospirillales) | Isolated from an anchialine pool on Hawaiian Islands, halophilic | (16) |
| *Alteromonadales* bacterium BS08 | Proteobacteria/Gammaproteobacteria/Alteromonadales | Marine bacteria | GenBank NZ_MRUG01000000 |
| *Teredinibacter turnerae* | Proteobacteria/Gammaproteobacteria/Cellvibrionales | Isolated from the gill tissue of a wood-boring mollusc (shipworm), marine isolate, cellulytic | (17) |
| *Saccharophagus degradans* | Proteobacteria/Gammaproteobacteria/Alteromonadales | Carbohydrate degrading marine bacteria | (18) |
| *Marinimicrobium agarilyticum* | Proteobacteria/Gammaproteobacteria/Cellvibrionales | Isolated from tidal flat sediment of the South Sea in Korea, halotolerant | (19) |
| *Motiliproteus* sp. MSK22-1 | Proteobacteria/Gammaproteobacteria/[Oceanospirillales](https://en.wikipedia.org/wiki/Oceanospirillales) | Marine bacteria, isolated from pacific ocean island | GenBank NZ_MIEQ01000000 |
| *Marinobacterium stanieri* | Proteobacteria/Gammaproteobacteria/[Oceanospirillales](https://en.wikipedia.org/wiki/Oceanospirillales) | Marine bacterium | (14) |
| *Paraliobacillus ryukyuensis* | Firmicutes/Bacilli/Bacillales | Isolated from a decomposing marine alga collected in Okinawa, Japan, halophilic, extremely halotolerant, alkaliphilic | (20) |
| *Candidatus thiodiazotropha endolucinida* | Proteobacteria/Gammaproteobacteria/unclassified | Endosymbiont of the mollusk [Codakia orbicularis, sulfur oxidizing, nitrogen fixation](https://www.google.es/url?sa=t&rct=j&q=&esrc=s&source=web&cd=1&cad=rja&uact=8&ved=0ahUKEwj67se3-53aAhVi0aYKHROgAxIQFggqMAA&url=https%3A%2F%2Fes.wikipedia.org%2Fwiki%2FCodakia_orbicularis&usg=AOvVaw2I3YJR5TSDmLLEa0Z1XDG4) | (21) |
| *Arhodomonas aquaeolei* | Proteobacteria/Gammaproteobacteria/[Chromatiales](https://en.wikipedia.org/wiki/Chromatiales) | Isolated from subterranean brine, halophilic | (22) |
| *Sedimenticola selenatireducens* | Proteobacteria/Gammaproteobacteria/unclassified | Isolated from sediment samples of inter-tidal regions, selenate respiring | (23) |
| *Dissulfuribacter thermophilus* | Proteobacteria/Deltaproteobacteria/unclassified | Isolated from a deep-sea hydrothermal vent chimney in the Pacific ocean, uses elemental sulfur as the only energy source | (24) |
| *Desulfarculus baarsii* | Proteobacteria/Deltaproteobacteria/[Desulfarculales](https://de.wikipedia.org/wiki/Desulfarculales) | Sulfate reducing bacterium, isolated from ditch sediment near Konstanz University | (25) |
| *Desulfocarbo indianensis* | Proteobacteria/Deltaproteobacteria/[Desulfarculales](https://de.wikipedia.org/wiki/Desulfarculales) | Sulfate-reducing, isolated from water extracted from a coal bed in Indiana, USA | (26) |
| *Malonomonas rubra* | Proteobacteria/Deltaproteobacteria/[Desulfuromonadales](https://en.wikipedia.org/wiki/Desulfuromonadales) | Isolated from anoxic marine sediment samples, grows on malonate | (27) |
| *Methanocaldococcus villosus* | [Euryarchaeota](https://es.wikipedia.org/wiki/Euryarchaeota) (Archaea)/[Methanococci](https://www.ncbi.nlm.nih.gov/Taxonomy/Browser/wwwtax.cgi?mode=Undef&id=183939&lvl=3&lin=f&keep=1&srchmode=1&unlock)/[Methanococcales](https://www.ncbi.nlm.nih.gov/Taxonomy/Browser/wwwtax.cgi?mode=Undef&id=2182&lvl=3&lin=f&keep=1&srchmode=1&unlock) | Isolated from a submarine hydrothermal system at the Kolbeinsey Ridge, north of Iceland, heavily flagellated | (28) |
| *Marinobacterium georgiense* | Proteobacteria/Gammaproteobacteria/[Oceanospirillales](https://www.ncbi.nlm.nih.gov/Taxonomy/Browser/wwwtax.cgi?mode=Undef&id=135619&lvl=3&lin=f&keep=1&srchmode=1&unlock) | Isolated from marine pulp mill effluent, cellulytic | (29) |
| *Candidatus Scalindua rubra* | [Planctomycetes](https://en.wikipedia.org/wiki/Planctomycetes)/[Planctomycetia](https://www.ncbi.nlm.nih.gov/Taxonomy/Browser/wwwtax.cgi?mode=Undef&id=203683&lvl=3&lin=f&keep=1&srchmode=1&unlock)/[Candidatus Brocadiales](https://www.ncbi.nlm.nih.gov/Taxonomy/Browser/wwwtax.cgi?mode=Undef&id=1127829&lvl=3&lin=f&keep=1&srchmode=1&unlock) | Interface Above the Discovery Deep Brine in the Red Sea, | (30) |
| *Sulfurospirillum* sp. UBA12182 | Proteobacteria/Epsilonprotobacteria/Campylobacterales | Able to oxidize elemental sulfur | (31) |
| *Beggiatoa* sp. 4572_84 | Proteobacteria/Gammaproteobacteria/Thiotrichales | Sulfide oxidizers, found in coastal sediments, can store nitrate | (32) |
| *Candidatus Thiomargarita nelsonii* | Proteobacteria/Gammaproteobacteria/[Thiotrichales](https://en.wikipedia.org/wiki/Thiotrichales) | Sulfur and sulfide oxidizers | (33) |
| *Sulfurovum* sp. NBC37-1 | Proteobacteria/Epsilonprotobacteria/Unclassified | Sulfur oxidizing bacteria, isolated from sediment of the Iheya North hydrothermal system in the mid-Okinawa Trough, Japan | (34) |
| *Thiotrichales* bacterium HS_08 | Proteobacteria/Gammaproteobacteria/Thiotrichales | Sulfur oxidizing bacteria | GenBank NZ_FMSV02000000 |
| *Thermopetrobacter* sp. TC1 | Proteobacteria/Alphaproteobacteria/unclassified | Probably sulfur oxidizing bacterium | (35) |
| *Campylobacteraceae* bacterium 4484_4 | Proteobacteria/Epsilonprotobacteria/Campylobacteriales | Multiple lifestyles | GenBank NZ_JQKX01000000 |
| *Sulfitobacter pseudonitzschiae* | Proteobacteria/Alphaproteobacteria/[Rhodobacterales](https://www.ncbi.nlm.nih.gov/Taxonomy/Browser/wwwtax.cgi?mode=Undef&id=204455&lvl=3&keep=1&srchmode=1&unlock) | Isolated from the toxic marine diatom Pseudo-nitzschia multiseries, able to reduce nitrate and oxidize sulfite | (36) |
| *Elusimicrobia* bacterium GWA2_66_18 | Elusimicrobia/Elusimicrobi**/[Elusimicrobiales](https://www.ncbi.nlm.nih.gov/Taxonomy/Browser/wwwtax.cgi?mode=Tree&id=641854&lvl=3&lin=f&keep=1&srchmode=1&unlock)** | Isolated from the hindgut of the termite Reticulitermes speratus, but widespread in the environment | (37) |

**References**

1. Li Y, Zhou M, Wang F, Wang ET, Du Z, Wu C, Zhang Z, Liu W, Xie Z. 2017. *Photobacterium proteolyticum* sp. nov., a protease-producing bacterium isolated from ocean sediments of Laizhou Bay. Int J Syst Evol Microbiol 67:1835-1840.

2. Lauro FM, Chastain RA, Ferriera S, Johnson J, Yayanos AA, Bartlett DH. 2013. Draft Genome Sequence of the Deep-Sea Bacterium *Shewanella benthica* Strain KT99. Genome Announc 1.

3. Nogi Y, Hosoya S, Kato C, Horikoshi K. 2007. *Psychromonas hadalis* sp. nov., a novel piezophilic bacterium isolated from the bottom of the Japan Trench. Int J Syst Evol Microbiol 57:1360-4.

4. Bagheri M, Amoozegar MA, Didari M, Makhdoumi-Kakhki A, Schumann P, Sproer C, Sanchez-Porro C, Ventosa A. 2013. *Marinobacter persicus* sp. nov., a moderately halophilic bacterium from a saline lake in Iran. Antonie Van Leeuwenhoek 104:47-54.

5. Weiser JN, Ferreira DM, Paton JC. 2018. *Streptococcus pneumoniae*: transmission, colonization and invasion. Nat Rev Microbiol doi:10.1038/s41579-018-0001-8.

6. Kordjian HH, Schultz JD, Rosenvinge FS, Moller J, Pedersen RM. 2015. First clinical description of *Eggerthia catenaformis* bacteremia in a patient with dental abscess. Anaerobe 35:38-40.

7. Davin-Regli A, Pages JM. 2015. *Enterobacter aerogenes* and *Enterobacter cloacae*; versatile bacterial pathogens confronting antibiotic treatment. Front Microbiol 6:392.

8. Satomi M, Kimura B, Hayashi M, Shouzen Y, Okuzumi M, Fujii T. 1998. *Marinospirillum* gen. nov., with descriptions of *Marinospirillum megaterium* sp. nov., isolated from kusaya gravy, and transfer of *Oceanospirillum minutulum* to *Marinospirillum minutulum* comb. nov. Int J Syst Bacteriol 48 Pt 4:1341-8.

9. Satomi M, Kimura B, Hayashi M, Okuzumi M, Fujii T. 2004. *Marinospirillum insulare* sp. nov., a novel halophilic helical bacterium isolated from kusaya gravy. Int J Syst Evol Microbiol 54:163-7.

10. Zhang W, Xue Y, Ma Y, Grant WD, Ventosa A, Zhou P. 2002. *Marinospirillum alkaliphilum* sp. nov., a new alkaliphilic helical bacterium from Haoji soda lake in Inner Mongolia Autonomous Region of China. Extremophiles 6:33-7.

11. Namsaraev Z, Akimov V, Tsapin A, Barinova E, Nealson K, Gorlenko V. 2009. *Marinospirillum* celere sp. nov., a novel haloalkaliphilic, helical bacterium isolated from Mono Lake. Int J Syst Evol Microbiol 59:2329-32.

12. Nakagawa T, Iino T, Suzuki K, Harayama S. 2006. *Ferrimonas futtsuensis* sp. nov. and *Ferrimonas kyonanensis* sp. nov., selenate-reducing bacteria belonging to the Gammaproteobacteria isolated from Tokyo Bay. Int J Syst Evol Microbiol 56:2639-45.

13. Sidhu C, Thakur S, Sharma G, Tanuku NRS, Pinnaka AK. 2017. *Oceanospirillum sanctuarii* sp. nov., isolated from a sediment sample. Int J Syst Evol Microbiol 67:3428-3434.

14. Satomi M, Kimura B, Hamada T, Harayama S, Fujii T. 2002. Phylogenetic study of the genus Oceanospirillum based on 16S rRNA and *gyr*B genes: emended description of the genus *Oceanospirillum*, description of *Pseudospirillum* gen. nov., *Oceanobacter* gen. nov. and *Terasakiella* gen. nov. and transfer of *Oceanospirillum jannaschii* and *Pseudomonas stanieri* to *Marinobacterium* as *Marinobacterium jannaschii* comb. nov. and *Marinobacterium stanieri* comb. no. Int J Syst Evol Microbiol 52:739-47.

15. Arahal DR, Lekunberri I, Gonzalez JM, Pascual J, Pujalte MJ, Pedros-Alio C, Pinhassi J. 2007. *Neptuniibacter caesariensis* gen. nov., sp. nov., a novel marine genome-sequenced gammaproteobacterium. Int J Syst Evol Microbiol 57:1000-6.

16. Zepeda VK, Busse HJ, Golke J, Saw JH, Alam M, Donachie SP. 2015. *Terasakiispira papahanaumokuakeensis* gen. nov., sp. nov., a gammaproteobacterium from Pearl and Hermes Atoll, Northwestern Hawaiian Islands. Int J Syst Evol Microbiol 65:3609-17.

17. Distel DL, Morrill W, MacLaren-Toussaint N, Franks D, Waterbury J. 2002. *Teredinibacter turnerae* gen. nov., sp. nov., a dinitrogen-fixing, cellulolytic, endosymbiotic gamma-proteobacterium isolated from the gills of wood-boring molluscs (Bivalvia: Teredinidae). Int J Syst Evol Microbiol 52:2261-9.

18. Ekborg NA, Gonzalez JM, Howard MB, Taylor LE, Hutcheson SW, Weiner RM. 2005. *Saccharophagus degradans* gen. nov., sp. nov., a versatile marine degrader of complex polysaccharides. Int J Syst Evol Microbiol 55:1545-9.

19. Lim JM, Jeon CO, Lee JC, Song SM, Kim KY, Kim CJ. 2006. *Marinimicrobium koreense* gen. nov., sp. nov. and *Marinimicrobium agarilyticum* sp. nov., novel moderately halotolerant bacteria isolated from tidal flat sediment in Korea. Int J Syst Evol Microbiol 56:653-7.

20. Ishikawa M, Ishizaki S, Yamamoto Y, Yamasato K. 2002. *Paraliobacillus ryukyuensis* gen. nov., sp. nov., a new Gram-positive, slightly halophilic, extremely halotolerant, facultative anaerobe isolated from a decomposing marine alga. J Gen Appl Microbiol 48:269-79.

21. Konig S, Gros O, Heiden SE, Hinzke T, Thurmer A, Poehlein A, Meyer S, Vatin M, Mbeguie AMD, Tocny J, Ponnudurai R, Daniel R, Becher D, Schweder T, Markert S. 2016. Nitrogen fixation in a chemoautotrophic lucinid symbiosis. Nat Microbiol 2:16193.

22. Adkins JP, Madigan MT, Mandelco L, Woese CR, Tanner RS. 1993. *Arhodomonas aquaeolei* gen. nov., sp. nov., an aerobic, halophilic bacterium isolated from a subterranean brine. Int J Syst Bacteriol 43:514-20.

23. Narasingarao P, Haggblom MM. 2006. *Sedimenticola selenatireducen*s, gen. nov., sp. nov., an anaerobic selenate-respiring bacterium isolated from estuarine sediment. Syst Appl Microbiol 29:382-8.

24. Slobodkin AI, Reysenbach AL, Slobodkina GB, Kolganova TV, Kostrikina NA, Bonch-Osmolovskaya EA. 2013. *Dissulfuribacter thermophilus* gen. nov., sp. nov., a thermophilic, autotrophic, sulfur-disproportionating, deeply branching deltaproteobacterium from a deep-sea hydrothermal vent. Int J Syst Evol Microbiol 63:1967-71.

25. Sun H, Spring S, Lapidus A, Davenport K, Del Rio TG, Tice H, Nolan M, Copeland A, Cheng JF, Lucas S, Tapia R, Goodwin L, Pitluck S, Ivanova N, Pagani I, Mavromatis K, Ovchinnikova G, Pati A, Chen A, Palaniappan K, Hauser L, Chang YJ, Jeffries CD, Detter JC, Han C, Rohde M, Brambilla E, Goker M, Woyke T, Bristow J, Eisen JA, Markowitz V, Hugenholtz P, Kyrpides NC, Klenk HP, Land M. 2010. Complete genome sequence of *Desulfarculus baarsii* type strain (2st14). Stand Genomic Sci 3:276-84.

26. An TT, Picardal FW. 2014. *Desulfocarbo indianensis* gen. nov., sp. nov., a benzoate-oxidizing, sulfate-reducing bacterium isolated from water extracted from a coal bed. Int J Syst Evol Microbiol 64:2907-14.

27. Dehning I, Schink B. 1989. *Malonomonas rubra* gen. nov. sp. nov., a microaerotolerant anaerobic bacterium growing by decarboxylation of malonate. Arch Microbiol 151:427-433.

28. Bellack A, Huber H, Rachel R, Wanner G, Wirth R. 2011. *Methanocaldococcus villosus* sp. nov., a heavily flagellated archaeon that adheres to surfaces and forms cell-cell contacts. Int J Syst Evol Microbiol 61:1239-45.

29. Gonzalez JM, Mayer F, Moran MA, Hodson RE, Whitman WB. 1997. *Microbulbifer hydrolyticus* gen. nov., sp. nov., and *Marinobacterium georgiense* gen. nov., sp. nov., two marine bacteria from a lignin-rich pulp mill waste enrichment community. Int J Syst Bacteriol 47:369-76.

30. Speth DR, Lagkouvardos I, Wang Y, Qian PY, Dutilh BE, Jetten MSM. 2017. Draft Genome of *Scalindua rubra*, Obtained from the Interface Above the Discovery Deep Brine in the Red Sea, Sheds Light on Potential Salt Adaptation Strategies in Anammox Bacteria. Microb Ecol 74:1-5.

31. Schubert T. 2017. The organohalide-respiring bacterium *Sulfurospirillum multivorans*: a natural source for unusual cobamides. World J Microbiol Biotechnol 33:93.

32. Preisler A, de Beer D, Lichtschlag A, Lavik G, Boetius A, Jorgensen BB. 2007. Biological and chemical sulfide oxidation in a *Beggiatoa* inhabited marine sediment. ISME J 1:341-53.

33. Flood BE, Fliss P, Jones DS, Dick GJ, Jain S, Kaster AK, Winkel M, Mussmann M, Bailey J. 2016. Single-Cell (Meta-)Genomics of a Dimorphic *Candidatus Thiomargarita nelsonii* Reveals Genomic Plasticity. Front Microbiol 7:603.

34. Inagaki F, Takai K, Nealson KH, Horikoshi K. 2004. *Sulfurovum lithotrophicum* gen. nov., sp. nov., a novel sulfur-oxidizing chemolithoautotroph within the epsilon-Proteobacteria isolated from Okinawa Trough hydrothermal sediments. Int J Syst Evol Microbiol 54:1477-82.

35. Watanabe T, Kojima H, Fukui M. 2016. Identity of major sulfur-cycle prokaryotes in freshwater lake ecosystems revealed by a comprehensive phylogenetic study of the dissimilatory adenylylsulfate reductase. Sci Rep 6:36262.

36. Hong Z, Lai Q, Luo Q, Jiang S, Zhu R, Liang J, Gao Y. 2015. *Sulfitobacter pseudonitzschiae* sp. nov., isolated from the toxic marine diatom *Pseudo-nitzschia* multiseries. Int J Syst Evol Microbiol 65:95-100.

37. Herlemann DP, Geissinger O, Brune A. 2007. The termite group I phylum is highly diverse and widespread in the environment. Appl Environ Microbiol 73:6682-5.
