# Supplementary material for: The Molecular Mechanism of Nitrate Chemotaxis via Direct Ligand Binding to the PilJ Domain of McpN
Source: mBio. 2019 Feb 19;10(1):e02334-18. doi: 10.1128/mBio.02334-18 (PMC6381276; doi:10.1128/mBio.02334-18)
Supplement: TABLE S2 [file mBio.02334-18-st002.docx]

**Table S2)**

| Oligonucleotide | Sequence (5’-3’)^a^ | Purpose |
| --- | --- | --- |
| PA2788-NdeI-F | GGAATTCCATATGTACCTGAGCATGTCGATCTC | Generation of pMcpN-LBD |
| PA2788-XhoI-R | CCGCTCGAGTCACCACATCTGGGTGTGCTG |  |
| PA4520-NdeI-F | GGAATTCCATATGTATCTGGTGCGCGATGCCTA | Generation of pET4520-LBD |
| PA4520-SalI-R | TAATGTCGACTCACATCTGCGTGCGCACCT |  |
| PA0411-NdeI-F | GGAATTCCATATGAACTTTGCCTACCTCAACACC | Generation of pPilJ-LBD |
| PA0411-EcoRI-R | GGAATTCTCAGGCGTAACCGGCGAACA |  |
| q2788-F | AGGGGTTGATCGAGCAGTTG | RT-qPCR of the *mcpN* gene |
| q2788-R | TTGAGGCCGTCCAGTTCTTC |  |
| qrpoD-F | AGAAGAAAGCGACGACAGCA | RT-qPCR of the *rpoD* gene |
| qrpoD-R | CTTCTTGGCCTTGTCGAGCT |  |
| PA2788R61A-F: | CGTGGCCGGCGCGCAGGCCATGCTCAGCCAGAAGATGGC | Generation of pMcpN-R61A, overlapping PCR primers. |
| PA2788R61A-R: | CTTCTGGCTGAGCATGGCCTGCGCGCCGGCCACGTTGATG |  |
| 2788c_NdeI-F | taatcatATGAACGAAAGCGTCGCCAG | Generation of pBBRMcpN |
| 2788c_BamHI-R | taatggatccTCAGGTACGGAAGCGGCC |  |

^a^Underlined are the restriction enzyme targets. Highlighted in grey is the mutated codon.
