# Supplementary material for: The Molecular Mechanism of Nitrate Chemotaxis via Direct Ligand Binding to the PilJ Domain of McpN
Source: mBio. 2019 Feb 19;10(1):e02334-18. doi: 10.1128/mBio.02334-18 (PMC6381276; doi:10.1128/mBio.02334-18)
Supplement: TABLE S3 [file mBio.02334-18-st003.docx]

**Table S3)**

| Protein | McpN-LBD |
| --- | --- |
| Ligand | nitrate |
| PDB identifier | 6GCV |
| **Data collection** |  |
| Beam line | ID23-1 (ESRF) |
| Space Group | C 1 2 1 |
| Cell dimensions |  |
| a, b, c (Å)  β (^o^) | 92.78, 87.95, 52.89  93.05 |
| ASU | 2 |
| Resolution (Å) | 46.32 - 1.30  (1.35 - 1.30) |
| R*_merge_* (%) | 3.2 (37.4) |
| I/σ_I_ | 14.98 (2.38) |
| Completeness (%) | 85.43 (92.43) |
| Unique reflections | 88758 (9541) |
| Multiplicity | 2.7 (2.7) |
| CC(1/2) | 0.999 (0.837) |
| CC^*^ | 1 (0.955) |
| **Refinement** |  |
| Resolution (Å) | 46.32 - 1.30 |
| R*_work_*/R*_free_* (%) | 12.53 / 15.32 |
| CC(wok)/CC(free) (%) | 97.2 / 96.1 |
| No. atoms | 3683 |
| Protein | 3217 |
| Ligands | 36 |
| Water | 430 |
| B-factor (Å^2^) | 17.09 |
| R.m.s deviations |  |
| Bond lengths (Å) | 0.013 |
| Bond angles (^0^) | 1.41 |
| Ramachandran (%) |  |
| Favored | 100 |
| Outliers | 0 |
| Average B-factor | 24.51 |
| macromolecules | 22.96 |
| ligands | 32.60 |
| solvent | 35.42 |
